# DNA methylation at an enhancer of the three prime repair exonuclease 2 gene (*TREX2*) is linked to gene expression and survival in laryngeal cancer

**DOI:** 10.1186/s13148-019-0666-5

**Published:** 2019-05-03

**Authors:** Christoph Weigel, Jittiporn Chaisaingmongkol, Yassen Assenov, Christine Kuhmann, Volker Winkler, Irene Santi, Olga Bogatyrova, Simone Kaucher, Justo L. Bermejo, Suet Y. Leung, Tsun L. Chan, Felix Lasitschka, Manfred H. Bohrer, Alexander Marx, Roland Heyny-von Haußen, Christel Herold-Mende, Gerhard Dyckhoff, Petra Boukamp, Klaus W. Delank, Karl Hörmann, Burkhard M. Lippert, Gerald Baier, Andreas Dietz, Christopher C. Oakes, Christoph Plass, Heiko Becher, Peter Schmezer, Heribert Ramroth, Odilia Popanda

**Affiliations:** 1 Division of Cancer Epigenomics, German Cancer Research Center (DKFZ), Im Neuenheimer Feld 280, 69120 Heidelberg, Germany; 2 Chulabhorn Research Institute, Bangkok, Thailand; 3 Institute of Public Health, University of Heidelberg, Heidelberg, Germany; 4 Institute of Medical Biometry and Informatics, University Hospital Heidelberg, Heidelberg, Germany; 5 Department of Pathology, The University of Hong Kong, Queen Mary Hospital, Pokfulam, Hong Kong, China; 6 Department of Pathology, Hong Kong Sanatorium and Hospital, Happy Valley, Hong Kong, China; 7 Institute of Pathology, University Hospital Heidelberg, Heidelberg, Germany; 8 Medical Hospital, Institute of Pathology, Ludwigshafen, Germany; 9 Institute of Pathology, University Medical Centre Mannheim, Mannheim, Germany; 10 Center of Pathology, Klinikum Darmstadt, Darmstadt, Germany; 11 Department of Otorhinolaryngology, Head and Neck Surgery, University of Heidelberg, Heidelberg, Germany; 12 Division of Neurosurgical Research, Neurosurgery, University of Heidelberg, Heidelberg, Germany; 13 Division of Genetics of Skin Carcinogenesis, German Cancer Research Center (DKFZ), Heidelberg, Germany; 14 Institute for Environmental Medicine, IUF, Düsseldorf, Germany; 15 Medical Hospital, Head and Neck Surgery, Ludwigshafen, Germany; 16 Department of Otorhinolaryngology, Head and Neck Surgery, University Hospital of Mannheim, Mannheim, Germany; 17 Department of Otorhinolaryngology, Head and Neck Surgery, Heilbronn, Germany; 18 Department of Otorhinolaryngology, Head and Neck Surgery, Academic Teaching Hospital, Darmstadt, Germany; 19 Department of Otorhinolaryngology, Head and Neck Surgery, University of Leipzig, Leipzig, Germany; 20 German Cancer Research Consortium (DKTK), Heidelberg, Germany; 21 Institute of Medical Biometry and Epidemiology, University Medical Center Hamburg-Eppendorf, Hamburg, Germany; 22 Present Address: Division of Hematology Department of Internal Medicine, The Ohio State University, Columbus, OH USA

**Keywords:** Epigenetics, DNA repair, DNA methylation, *TREX2*, Pan-cancer studies, TCGA, Laryngeal cancer, Head and neck cancer, HNSCC, Patient survival

## Abstract

**Background:**

Genetic aberrations in DNA repair genes are linked to cancer, but less is reported about epigenetic regulation of DNA repair and functional consequences. We investigated the intragenic methylation loss at the three prime repair exonuclease 2 (*TREX2*) locus in laryngeal (*n* = 256) and colorectal cancer cases (*n* = 95) and in pan-cancer data from The Cancer Genome Atlas (TCGA).

**Results:**

Significant methylation loss at an intragenic site of *TREX2* was a frequent trait in both patient cohorts (*p* = 0.016 and < 0.001, respectively) and in 15 out of 22 TCGA studies. Methylation loss correlated with immunohistochemically staining for TREX2 (*p* < 0.0001) in laryngeal tumors and improved overall survival of laryngeal cancer patients (*p* = 0.045). Chromatin immunoprecipitation, demethylation experiments, and reporter gene assays revealed that the region of methylation loss can function as a CCAAT/enhancer binding protein alpha (CEBPA)-responsive enhancer element regulating *TREX2* expression.

**Conclusions:**

The data highlight a regulatory role of *TREX2* DNA methylation for gene expression which might affect incidence and survival of laryngeal cancer. Altered TREX2 protein levels in tumors may affect drug-induced DNA damage repair and provide new tailored therapies.

**Electronic supplementary material:**

The online version of this article (10.1186/s13148-019-0666-5) contains supplementary material, which is available to authorized users.

## Background

Exposure to genotoxic agents during smoking [1] and alcohol consumption [2], as well as by workplace hazards [3, 4], is linked to cancer incidence, as shown for laryngeal cancer [2], head and neck squamous cell carcinoma (HNSCC), and also colorectal cancer (CRC) [5]. Cancer incidence and treatment response, however, are highly diverse among patients, despite similar carcinogenic exposures or treatment options [6]. Recent research has highlighted the importance of genetic variation in DNA repair and tumor suppressor genes for the response to genotoxic exposure and cancer risk [5, 7, 8], but genetic variants alone cannot fully explain the heterogeneous treatment and survival outcomes observed [8, 9]. Epigenetic traits such as DNA methylation patterns have emerged as further determinants of cancer incidence and outcome by silencing promoters of DNA repair and tumor suppressor genes [10–15]. Methylation of gene enhancers is also involved in cell type-specific gene activation or repression by controlling transcription factor binding sites [16]. Individual gene enhancers and their epigenetic regulation in diseases are, however, still poorly understood, but experimental evidence points to a substantial role of DNA methylation [17].

Recently, we identified DNA methylation changes at promoter regions of DNA repair genes in HNSCC and other tumors using quantitative methylation analysis [18]. Yet, the molecular function of DNA methylation at the affected gene loci has remained unexplored. We here quantified DNA methylation at the DNA repair gene three prime repair exonuclease 2 (*TREX2*) in tumor tissue compared to adjacent normal tissue in an independent, population-based case-control study of laryngeal cancer patients from Germany [3, 7]. *TREX2* is a gene recently reported to be involved in mutagen-induced skin and oral carcinogenesis [19, 20] and DNA repair [21, 22] and might thus also be linked to the etiology of laryngeal cancer. We observed loss of DNA methylation at a *TREX2* intragenic gene locus in laryngeal cancer, colorectal cancer, and further cancer studies from The Cancer Genome Atlas (TCGA). Decreased *TREX2* DNA methylation was associated with elevated TREX2 expression and CCAAT/enhancer binding protein alpha (CEBPA)-mediated regulation in vitro. Low *TREX2* methylation correlated with prolonged overall survival in laryngeal and colorectal cancer. In summary, epigenetic deregulation of *TREX2* expression was observed in multiple cancers. This highlights its potential involvement in fundamental cellular responses to tumorigenesis.

## Results

### Reduced DNA methylation of *TREX2* in laryngeal cancer

**Fig. 1:**
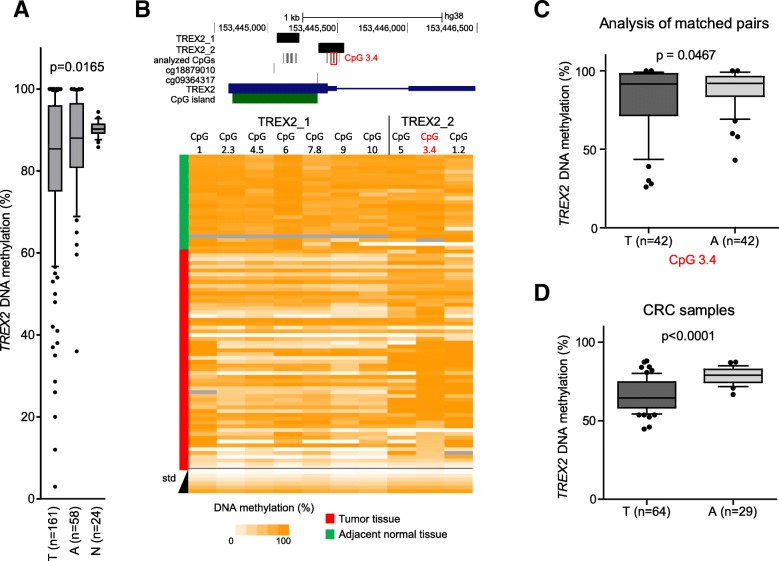
Identification of differential methylation of the *TREX2* gene in laryngeal cancer. **a** Quantitative DNA methylation analysis using EpiTYPER assay in a cohort of 161 laryngeal cancer tumor tissues (T), 58 adjacent non-cancerous normal tissues (A), and 24 normal mucosa samples from non-cancer patients who underwent tonsillectomy (N). Average methylation data for EpiTYPER TREX2_2 amplicon (see Fig. 1b) are shown; the *p* value refers to ANOVA test across the three sample subsets. **b** Upper panel: map of the *TREX2* gene locus with EpiTYPER PCR amplicons, single CpG dinucleotides analyzed in EpiTYPER (black), Illumina Infinium BeadChIP CpG probe locations (cg18879010 and cg 09364317), *TREX2* transcript (blue), and CpG islands (green) indicated. Informative CpG unit 3.4 of TREX2_2 is marked with a red box. Lower panel: heat map showing EpiTYPER results for amplicons TREX2_1 and TREX2_2 in laryngeal cancer tumors (*n* = 58, red) and adjacent normal tissue controls (*n* = 25, green), with DNA methylation at individual CpGs depicted as a color gradient ranging from white (non-methylated) to orange (fully methylated). Corresponding EpiTYPER amplicons are listed above, and DNA methylation standard values (std) are shown below. Grey: data unavailable/excluded. **c** Methylation analysis of matched pairs of laryngeal tumor and adjacent tissue samples at the informative CpG unit 3.4 of the *TREX2_2* amplicon; *p* value for two-tailed Student’s unpaired *t* test. **d** Average *TREX2*_2 methylation in colorectal cancer tumor tissues (T) and adjacent normal tissues (**a**). *p* value for two-tailed Student’s paired *t* test. Box-whisker plots show mean with 10 to 90 percentile

DNA methylation of the *TREX2* gene was measured in formalin-fixed paraffin-embedded (FFPE) tumor (*n* = 181) and adjacent non-tumor tissue samples (*n* = 75) from the German laryngeal cancer study (Fig. 1a, Table 1). Amplification from bisulfite-treated DNA and the quantitative EpiTYPER assay were used [23]. Methylation analysis focused on a region covering the *TREX2*-related CpG island (Fig. 1b, upper panel) as DNA quality and amount were limited by the available FFPE tissue sections. Accuracy of the *TREX2* EpiTYPER assays was confirmed applying stringent quality controls for PCR and EpiTyper readout, and a set of artificially methylated DNA standards which showed a good correlation of observed to expected methylation values (Fig. 1b, lower panel). An additional set of mucosa samples obtained during tonsillectomy from donors without cancer (*n* = 24) served as additional non-cancerous control tissues. We found reduced DNA methylation in laryngeal tumor samples for *TREX2* (*p* = 0.0165) comparing methylation means of all tumor and control samples with successful methylation assays. In addition, a decrease in methylation was also detected in some adjacent non-tumor tissues. A more detailed analysis for single CpG sites of the investigated *TREX2* CpG island is shown in a subset of laryngeal cancer samples (*n* = 58) and adjacent non-tumor tissues (*n* = 25, Fig. 1b, lower panel). Pairwise analysis of a subset of matched tumor and adjacent normal tissues (*n* = 42) revealed again lower methylation in about 50% of tumor tissues, especially when the CpG unit 3.4 consisting of two CpG sites in the TREX2_2 amplicon (marked in red in Fig. 1b, upper level) was analyzed (*p* = 0.0467, Fig. 1c).

**Table 1:** Demographic, clinical, and environmental exposure characteristics of cancer patients from the German laryngeal cancer study

Parameters	Category	Patients with tumor tissue, *N* (%)	Median follow-up time^1^, years	Patients with adjacent normal tissue, *N* (%)	Median follow-up time^1^, years
Total		181 (100)	8.5	75 (100)	8.2
Vital status^2^	Deceased	112 (61.9)	4.8	46 (61.3)	4.1
	Censored	64 (35.4)	12.0	28 (37.3)	12.2
Gender	Male	167 (92.3)	8.3	72 (96.0)	8.1
	Female	14 (7.7)	10.8	3 (4.0)	11.4
Age	Under 50	12 (6.6)	10.7	6 (8.0)	11.0
	50 to < 60	54 (29.8)	11.2	23 (30.7)	12.0
	60 to < 70	70 (38.7)	7.2	30 (40.0)	5.7
	Over 70	45 (24.9)	7.0	16 (21.3)	4.2
Tumor location	Glottic	111 (61.3)	10.3	47 (62.7)	9.2
	Supraglottic	48 (26.5)	5.4	19 (25.3)	8.1
	Subglottic	5 (2.8)	7.2	1 (1.3)	4.1
	Transglottic	11 (6.1)	5.0	7 (9.3)	2.1
	Unknown	6 (6.1)	–	1 (1.3)	–
Tumor stage^3^	I	68 (37.6)	10.8	29 (38.7)	8.4
	II	47 (26.0)	10.5	22 (29.3)	11.0
	III	21 (11.6)	6.3	11 (14.7)	8.1
	IV	38 (21.0)	3.5	12 (16.0)	5.1
Recurrences^3^	0	134 (74.0)	9.0	50 (66.7)	9.1
	1+	43 (27.8)	7.1	24 (32.0)	7.1
Second primary tumors^3^	0	151 (83.4)	9.1	64 (85.3)	9.4
	1+	25 (13.8)	5.4	10 (13.3)	3.8
Smoking (pack-years)	0	8 (4.4)	10.0	4 (5.3)	8.4
	≤ 20	21 (11.6)	11.6	9 (12.0)	11.8
	> 20 to ≤ 40	64 (35.4)	9.0	28 (37.3)	6.3
	> 40	88 (48.6)	7.5	34 (45.3)	8.7
Alcohol consumption (g ethanol/day)	≤ 25	53 (29.3)	10.1	21 (28.0)	11.0
	> 25 to ≤ 75	67 (37.0)	8.0	33 (44.0)	7.1
	> 75	61 (33.7)	8.6	21 (28.0)	8.0

^1^Median follow-up time since diagnosis

^2^Six patients are lost to follow-up (five patients with tumor tissue and one patient with adjacent normal tissue)

^3^Clinical records are missing for eight patients (seven patients with tumor tissue and one patient with adjacent normal tissue)

### *TREX2* DNA methylation loss as a frequent event in cancer

We further asked whether *TREX2* methylation loss can also be observed in other tumor types. Applying the EpiTYPER assay in a CRC patient cohort (64 and 29 adjacent normal tissues), we found significant *TREX2* methylation loss at the differentially methylated region initially identified in laryngeal cancer (Fig. 1d).

**Fig. 2:**
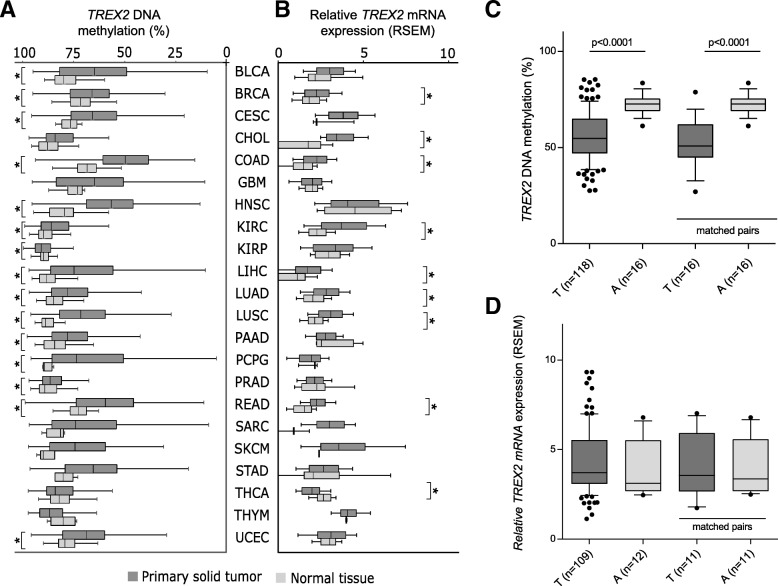
*TREX2* methylation loss as a frequent event in multiple cancer studies. **a** Differential DNA methylation and **b** mRNA expression of *TREX2* in multiple cancer studies (for abbreviations of cancer types, see Additional file 1: Table S1A). Graph shows **a** DNA methylation average at Illumina Infinium CpG probes cg09364317 and cg18879010 located at the TREX2 DMR and **b** TCGA Illumina HiSeq2000 RNAseq cohorts separated by primary tumor and normal tissue type. mRNA expression data depict RNA expression of the *TREX2* transcript as log2(*x* + 1) transformed RSEM-normalized count. *p < 0.05. In addition, laryngeal cancer patients, a subgroup of HNSC patients with the ICD10-code C32, were evaluated for overall and pairwise differences in *TREX2* DNA methylation (**c**) and mRNA expression (**d**)

In addition, we investigated *TREX2* DNA methylation in several TCGA cancer studies (Additional file 1: Table S1A). The TCGA methylome data were measured with Illumina Infinium 450K BeadChip arrays. The TREX2 locus is interrogated by seven CpG dinucleotide probes (Fig. 1b, Additional file 1: Figure S1). In the HNSCC patient cohort (*n* = 528) from TCGA, DNA methylation loss was found strongest for probes cg09364317 and cg18879010 and to a minor extent for cg12869875 and cg07206019 while nearby regions largely retained their high degree of methylation. The differentially methylated region (DMR) covered by probes cg09364317 and cg18879010 was scrutinized in the further TCGA cancer studies (Fig. 1b). Mean *TREX2* methylation was significantly reduced (*p* < 0.05) in 15 out of 22 (68%) cancer types (Fig. 2a, Additional file 1: Table S1B). Comparison of mean methylation values showed the strongest differences for head and neck cancer (HNSC; 24.5%), pheochromocytoma and paraganglioma (PCPG; 26.5%), colon adenocarcinoma (COAD; 20.2%), lung squamous cell carcinoma (LUSC; 18.5%), and liver hepatocellular carcinoma (LIHC; 19.2%). When laryngeal cancer patients were analyzed as a subgroup of HNSC patients, a significant reduction of mean methylation of 18.5% was observed (Fig. 2c). In a matched pair analysis, significant differential methylation was observed for HNSC, laryngeal cancer, COAD, LUSC, and other cancers (Additional file 1: Table S1C, Fig. 2c). Overall, this pan-cancer analysis suggests methylation loss at the TREX2 locus as a frequent event in cancer.

### TREX2 DNA methylation loss in tumors is associated with increased mRNA and protein expression

Methylation decrease at the *TREX2* DMR in tumor tissue should be associated with an increase in gene expression. As RNA quality obtained from FFPE tissue samples was insufficient, this association was analyzed in HNSCC samples from TCGA where methylation and RNA expression data were available. A significant inverse correlation between *TREX2* DMR methylation and *TREX2* mRNA expression was found, mainly for the cg09364317 probe and *TREX2* mRNA expression (*R* = − 0.143, *p* = 0.001; Additional file 1: Figure S2). Comparison of RNA expression data from further TCGA studies revealed higher expression of *TREX2* in seven cancer studies which showed also lower DNA methylation (Fig. 2a, b). This was not detected in the subgroup of laryngeal cancer patients which did not show differential TREX2 mRNA expression (Fig. 2c, d).

**Fig. 3:**
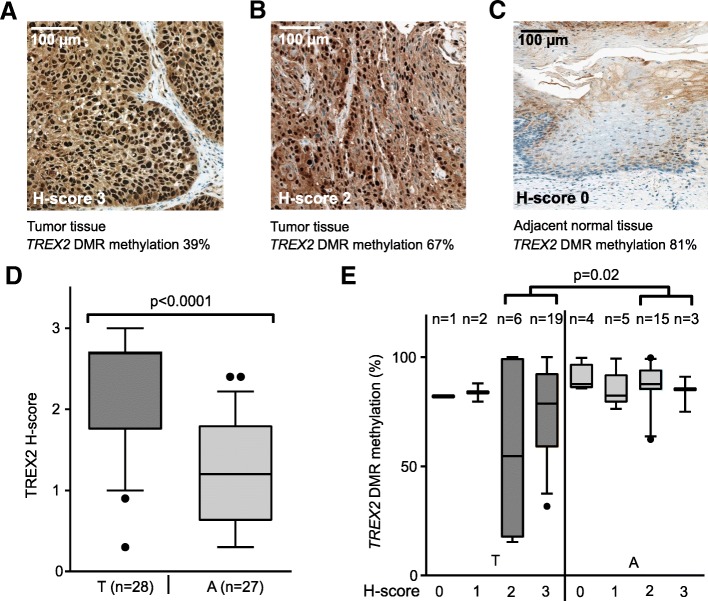
TREX2 protein expression in laryngeal cancer is associated with DNA methylation of the *TREX2* DMR. **a**–**c** Representative images of IHC staining for TREX2 in laryngeal tumor and adjacent non-tumor tissues with different degrees of *TREX2* DNA methylation. Images show two laryngeal tumor tissues with reduced *TREX2* methylation (**a**, **b**) and one adjacent normal tissue sample with high methylation (**c**). H-scores ranging from 0 (nuclear TREX2 very low) to 3 (nuclear TREX2 very high) are indicated. **d** TREX2 H-score in tumor (T) and adjacent normal (A) tissue samples. H-score is plotted as continuous variable based on evaluation of TREX2 staining in cell nuclei. **e** TREX2 H-scores and corresponding *TREX2* DMR methylation (amplicon TREX2_2, see Fig. 1b) in laryngeal tumor (T) and adjacent non-tumor tissue (A) samples. Box-whisker plots show mean and 10 to 90 percentile

Using immunohistochemistry (IHC), we measured TREX2 protein levels in laryngeal cancer and adjacent normal tissue samples representative for high and low *TREX2* DNA methylation. We observed TREX2 localization in the nuclei of laryngeal epithelial cells (Fig. 3a–c). Quantifying IHC staining by H-scores from 0 (very low) to 3 (strong), TREX2 protein amount was significantly (*p* < 0.001) increased in tumor tissue (Fig. 3d), and tumor samples with high H-scores showed significantly (*p* = 0.02) lower methylation of the *TREX2* DMR (Fig. 3e). These expression data support the role of DNA methylation at *TREX2* for regulating protein levels in laryngeal cancer.

### Differential DNA methylation of *TREX2* is associated with survival in laryngeal cancer

Based on the functional link of DNA methylation and gene expression, we investigated the possible association of *TREX2* DMR methylation with overall survival in our laryngeal cancer cohort. Clinical and demographic data including tobacco and alcohol consumption, tumor stage, and anatomical location were assessed in this cohort and summarized for both the entire cohort, as well as the subgroup for which adjacent normal tissue was available (Table 1). Taking DNA methylation as a continuous variable and adjusting for age and gender, an association of the specific CpG unit 3.4 in the EpiTYPER TREX2_2 amplicon with overall survival was found (hazard ratio (HR) 0.339, 95% confidence limits (Cl) 0.118–0.978, *p* = 0.045, Table 2; for Kaplan-Meier curves, see Additional file 1: Figure S3).

**Table 2:** *TREX2* DNA methylation in tumor tissue and overall survival of cancer patients. Samples from the German laryngeal cancer study and TCGA cancer patients with the ICD10-code C32 for laryngeal cancer are shown. For TCGA laryngeal cancer patients, overall survival was also analyzed in relation to *TREX2* mRNA expression (given as log_2_ (normalized expression + 1)). HRs with *p* values < 0.05 are presented in italic

Gene/CpG site in Amplicon TREX2_2 or on 450K array (TCGA studies)	Observations/events, *N*/*N*	Univariable analysis^a^		Adjusted for age and gender^a^	
		HR (95% CI)	*p* value	HR (95% CI)	*p* value
German larynx study					
CpG 1,2	181/112	0.713 (0.295–1.722)	0.4523	0.678 (0.278–1.654)	0.7289
CpG 3,4		*0.347 (0.120–1.002)*	*0.0505*	*0.339 (0.118–0.978)*	*0.0453*
CpG 5		1.197 (0.677–2.114)	0.5364	1.078 (0.606–1.918)	0.7984
Average		0.657 (0.237–1.822)	0.4193	0.561 (0.198–1.592)	0.2776
TCGA laryngeal cancer cohort					
cg09364317	119/52	*0.176 (0.032–0.980)*	*0.0473*	*0.106 (0.017–0.686)*	*0.0184*
cg18879010		0.146 (0.017–1.257)	0.0798	0.207 (0.022–1.935)	0.1673
*TREX2* mRNA expression	118/52	*0.766 (0.625–0.939)*	*0.0104*	*0.726 (0.589–0.895)*	*0.0027*

^a^Hazard ratios (HRs) and 95% confidence interval (CI) for continuous change of methylation after univariate analysis and adjusted for age and gender

Next, we validated the association of *TREX2* methylation status and survival in TCGA patient cohorts. CpG probes cg09364317 (Additional file 1: Table S2A) and cg18879010 (Additional file 1: Table S2B) were chosen as they showed the greatest variation in methylation and were located closest to the region that we analyzed with EpiTYPER. In the TCGA laryngeal cancer cases, an adjusted HR value of 0.106 (95% Cl = 0.017–0.686) was found for the probe cg09364317, supporting the results of the German laryngeal cancer study (Table 2). In addition, COAD and KIRP patients from TCGA showed a significant survival benefit (*p* = 0.044 and 0.031) with decreased *TREX2* DMR methylation in the unadjusted analysis (Additional file 1: Table S2A). For cg18879010, 7 out of 20 studies showed a significant association in the unadjusted analysis (Additional file 1: Table S2B). Finally, when we correlated TREX2 mRNA expression with the overall survival in the TCGA cancer studies, significant HRs were calculated for laryngeal cancer (HR = 0.726; CL = 0.589–0.895, *p* = 0.0027; Table 2) and CRC patients (Additional file 1: Table S3). In summary, survival benefits in laryngeal and colon cancer patients linked to *TREX2* DMR methylation loss imply a functional role of this region in tumorigenesis.

### The *TREX2* DMR displays gene enhancer characteristics in multiple cell types

**Fig. 4:**
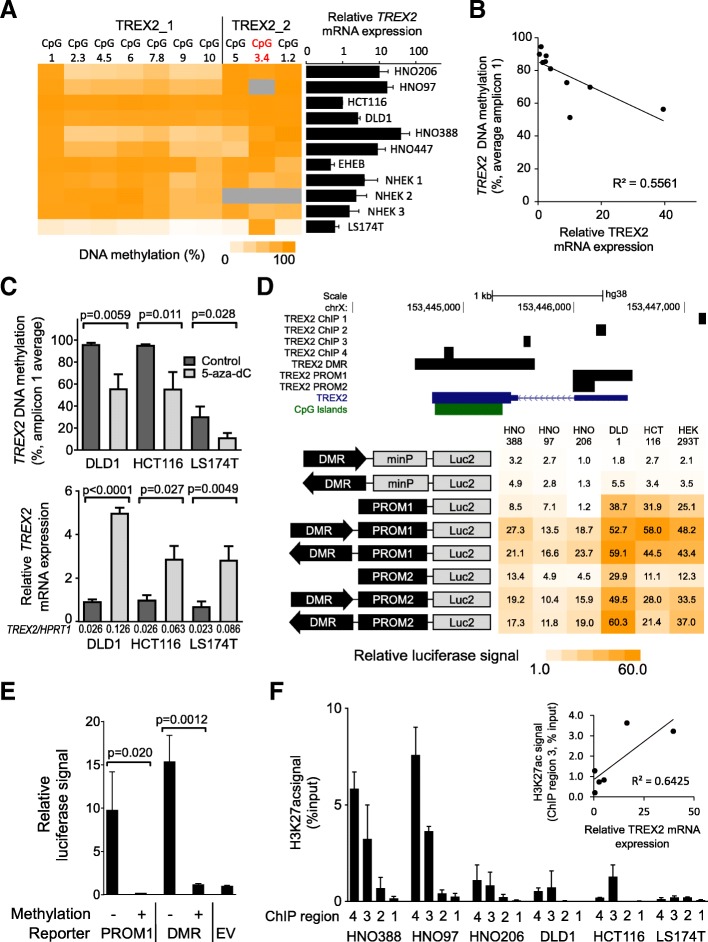
The *TREX2* DMR displays gene enhancer characteristics in multiple cell lines. **a** DNA methylation at individual CpG sites at the *TREX2* locus across different cancer cell lines and normal human epidermal keratinocyte (NHEK) specimens, matched with respective mRNA expression values (black bars). EHEB was included as a control with low *TREX2* expression. Dark gray: data unavailable. **b** Correlation of average DNA methylation at *TREX2*_1 EpiTYPER and *TREX2* mRNA expression determined by qRT-PCR in cell lines from **a**. LS174T was excluded due to reported X-chromosomal aberrations [24]. **c** 5-Aza-dC treatment in three cell lines and effects on *TREX2* DNA methylation and mRNA expression. Bar charts show cell lines after 6 days of treatment with 0.5 μM 5-aza-dC, depicting average TREX2 DNA methylation (upper panel) and *TREX2* mRNA expression (lower panel). Average expression ratios of *TREX2* versus the housekeeping gene *HPRT1* are depicted below each data point for approximate estimation of TREX2 mRNA abundance. **d** Upper panel: map of the *TREX2* gene with ChIP-qPCR amplicons and regions used for luciferase reporter assays. Lower panel: heat map depicting relative luciferase signals in cell lines transfected with *TREX2* reporter constructs. Schematic view of reporters is shown to the left. Signals depict mean of quadruplicate measurements normalized to empty vector (pGl4.23). minP/luc2, minimal promoter/luc2 luciferase. **e** HEK293T cells transfected with CpG-free reporter vectors carrying *TREX2* PROM1 and DMR (with minimal promoter) regions. Data show luciferase signal of in vitro CpG-methylated and non-methylated reporter plasmids. EV, empty vector (pCpGfree-promoter-lucia). **f** ChIP-qPCR assays at the *TREX2* locus. Bars show H3K27ac signal at four regions (see **d**) of the *TREX2* gene with mean and standard deviation from four replicates. Dot plot shows correlation of H3K27ac signal at the *TREX2* DMR (ChIP region 3) and relative *TREX2* mRNA expression in cell lines (*n* = 6). All data depict mean of three replicates with standard deviation, unless stated otherwise. *p* values refer to unpaired Student’s *t* test. For correlations, Pearson coefficient (*R*) is shown

To further investigate a functional link between *TREX2* methylation and expression, we screened eight cancer cell lines of different tissue origin and three normal human epidermal keratinocytes (NHEK) for a correlation between *TREX2* mRNA levels and methylation (Fig. 4a). DNA methylation patterns in CRC (HCT116, DLD1) and HNSCC cell lines (HNO216, HNO97, HNO388, HNO447) resembled the patterns of primary tumor samples especially for amplicon TREX2_1. Overall, expression and DNA methylation were inversely correlated (*R*^2^ = 0.5561, Fig. 4b). Low *TREX2* methylation and expression were found for cell line LS174T which carries an X-chromosomal deletion [24]. The DNA methyltransferase inhibitor 5-aza-2′-deoxycytidine (5-aza-dC) reduced *TREX2* DMR methylation and increased *TREX2* mRNA expression (Fig. 4c), supporting the possible regulatory role of the differentially methylated *TREX2* region for transcription. However, this observation did not clarify whether the *TREX2* DMR supported *TREX2* expression as a gene regulatory element or as a promoter site [25].

To identify regulatory properties across the *TREX2* locus, promoter and enhancer activities of *TREX2* regions were determined in a dual luciferase reporter assay. Using a reporter without promoter activity in ten different cell lines, the only region that induced a luciferase signal was a region located 3′ of the DMR covering a reported *TREX2* exon (TREX2_PROM1 and 2, Additional file 1: Figure S4A). The *TREX2* intragenic DMR was tested in sense and antisense orientation and did not show promoter activity (Additional file 1: Figure S4A, B). In line with this, transcription start site profiling via Cap analysis gene expression (CAGE)-seq from the FANTOM5 project [26] revealed a single *TREX2* transcription start site across various tissues which co-localizes with the promoter element TREX2_PROM1 (Additional file 1: Figure S5). Thus, we identified the *TREX2* promoter but excluded promoter activity for the *TREX2* DMR.

Further luciferase reporter assays combined the *TREX2* DMR with a minimal promoter or the endogenous *TREX2* promoter sequences TREX2_PROM1 and 2. The *TREX2* DMR increased activity of both promoters when added to the 5′ end of these sequences (Fig. 4d). In addition, the *TREX2* DMR acted in an orientation-independent manner, a feature of gene enhancers (Fig. 4d). In vitro CpG methylation of the luciferase reporters blocked the *TREX2* DMR activity (Fig. 4e), supporting a suppressive role of DNA methylation for this gene element.

Enhancer and promoter regions are characterized by specific chromatin modifications. Chromatin immunoprecipitation (ChIP) experiments revealed the presence of the enhancer marks histone H3 lysine 4 monomethylation (H3K4me1) and histone H3 lysine 27 acetylation (H3K27ac) at the *TREX2* DMR, supporting the classification of this region as a gene enhancer (Fig. 4f, Additional file 1: Figure S6). In addition, H3K27ac signals at the *TREX2* DMR correlated with *TREX2* mRNA expression (*R*^2^ = 0.6425, Fig. 4f). Taken together, these data suggest enhancer function for the *TREX2* DMR.

### *TREX2* DMR is activated by CEBPA

**Fig. 5:**
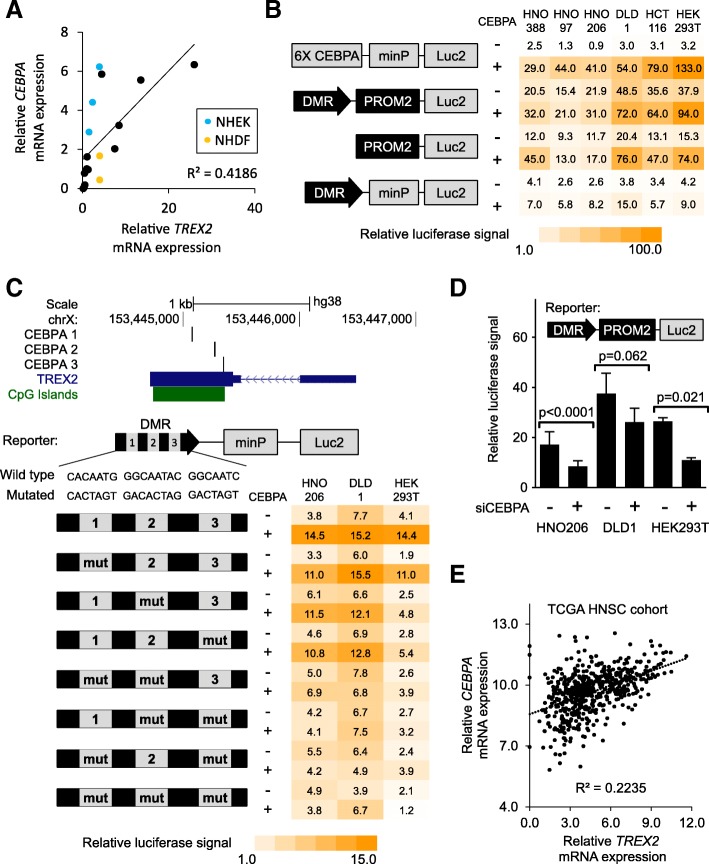
*TREX2* induction by the transcription factor CEBPA. **a** Correlation of average *TREX2* and *CEBPA* mRNA expression determined by qRT-PCR (duplicates) in cell lines and primary cells (*n* = 18); primary cells (NHEK, blue; NHDF, yellow) are marked. **b** Heat map depicting relative luciferase signal in different cell lines (HEK293T, colorectal cancer cells HCT116 and DLD1, HNSCC cells HNO388/97/206) transfected with *TREX2* luciferase reporter constructs depending on CEBPA levels. Schematic view of reporters is shown to the left. Luciferase signals depict mean of duplicates normalized to empty vector (pGl4.23) control. Co-transfection of CEBPA overexpression plasmid (CEBPA) is indicated. 6X CEBPA, synthetic CEBPA pathway reporter element with 6 tandem CEBPA consensus binding sites. **c** Upper panel: map of the *TREX2* gene locus with predicted CEBPA binding sites, *TREX2* transcript, and CpG islands are indicated. Lower panel: heat map depicting relative luciferase signal in different cell lines transfected with *TREX2* luciferase reporter constructs. Schematic view of reporters is shown to the left, with site-directed mutagenesis of predicted CEBPA binding sites (mut) indicated. Luciferase signals depict mean of duplicates normalized to empty vector (pGl4.23) control. Co-transfection of CEBPA overexpression plasmid (CEBPA) is indicated. **d** Luciferase reporter assay in different cell lines under co-treatment with siRNAs directed against *CEBPA* (siCEBPA). Schematic view of the transfected reporter construct (*TREX2* promoter and DMR) is included. Bars depict mean and standard deviation from quadruplicate experiments. **e** Correlation of *TREX2* and *CEBPA* mRNA expression determined by RNA sequencing in the TCGA head and neck squamous cell carcinoma (HNSC) cohort (*n* = 566), as log2(*x* + 1) transformed RSEM-normalized count. *p* values refer to unpaired Student’s *t* test. For correlations, Pearson coefficient (*R*) is shown. minP/Luc2, minimal promoter/luc2 luciferase gene included in the pGl4.23 vector

As the observed correlation of *TREX2* DMR methylation and mRNA expression was not very strong, we hypothesized that additional regulatory factors are required at the *TREX2* DMR to support gene expression. Transcription factors are candidates for such regulators [16]. We used sequence motif prediction tools to assess the potential binding of transcription factors at the *TREX2* DMR and promoter. Analysis focused on transcription factor binding motifs predicted by more than one algorithm (Additional file 1: Table S4) and those factors with the strongest correlation with mRNA *TREX2* expression in various cell lines and primary cells (*n* = 15, Additional file 1: Figure S7). Here, *CEBPA* mRNA expression correlated significantly with *TREX2* mRNA (*R*^2^ = 0.4186, 95% confidence interval 0.6465–3.029, *p* (slope non-zero) < 0.005; Fig. 5a). To assess the contribution of *CEBPA* to *TREX2* regulation in vitro, we overexpressed CEBPA and its closest protein family member CEBPB in CRC and HNSCC cell lines together with *TREX2* luciferase reporter constructs. Both CEBPA and CEBPB induced luciferase signals of the *TREX2* enhancer reporter (Fig. 5b, Additional file 1: Figure S8). The *TREX2* promoter, which also contains two conserved CEBPA binding sites, was also induced, and its activity was enhanced further by the addition of the TREX2 DMR in several of the tested cell lines. Mutating the predicted consensus CEBPA recognition sites reduced the CEBPA-induced *TREX2* enhancer (Fig. 5c), indicating that CEBPA motifs are directly involved in *TREX2* reporter activation. In cell lines with high *CEBPA* mRNA expression (HNO206 and DLD1), siRNA-mediated *CEBPA* knockdown significantly reduced *TREX2* DMR and promoter signals (Fig. 5d, Additional file 1: Figure S9). The affinity of CEBPA to predicted binding sites in the *TREX2* DMR and promoter regions was further confirmed in an in vitro proximity ligation assay (Additional file 1: Figure S10). Finally, RNA sequencing data from the TCGA HNSCC sample cohort were used to correlate *CEBPA* with *TREX2* mRNA levels (Fig. 5e). This correlation was significant (*R*^2^ = 0.2235, 95% confidence interval 0.2002–0.2732, *p* (slope non-zero) < 0.001), again indicating the activation of *TREX2* by CEBPA.

## Discussion

Using tumors and adjacent normal tissues from laryngeal cancer patients, we found DNA methylation loss at the *TREX2* locus for a substantial number of tumors which confirms the recently reported aberrant methylation in HNSCC [18]. Also other cancer entities such as colon adenocarcinoma showed similar differential methylation. *TREX2* DMR methylation was associated with altered protein and mRNA expression and improved survival in patients with laryngeal cancer from Germany and TCGA, suggesting a role of *TREX2* methylation in cancer etiology. The *TREX2* gene encodes a 3′ to 5′ exonuclease involved in DNA double-strand break repair [22]. *TREX2* knockout alters susceptibility to genotoxic agents in vivo and in vitro [19, 20]. In lingual epithelia and keratinocytes, TREX2 protein is involved in breakdown and degradation of DNA during differentiation and cornification [27, 28]. In cancers, recent data have indicated heterogeneous TREX2 levels caused by aberrant regulation. Rare genetic inactivation of *TREX2* has been reported in CRC [29], suggesting that TREX2 has a tumor suppressive function. In HNSCC and UV-exposed skin, TREX2 levels were shown to vary considerably, with high TREX2 being associated with enhanced UV protection and lower skin cancer risk [19]. Our data support literature data on the role of TREX2 in carcinogenesis as tumor patients with high TREX2 expression show improved overall survival in our analysis. Interestingly, this improved survival is not found when analyzing all TCGA HNSC cases (Additional file 1: Table S3), indicating that *TREX2* may have specific functions or regulation in laryngeal tissue distinct from other common sites of head and neck cancer.

There is additional evidence for the beneficial role of high TREX2 expression in tumors. A systematic analysis of *TREX2* expression in mice showed *TREX2* being most abundant in stratified epithelial tissues [20]. In our pan-cancer analysis of TCGA data, tumors with the strongest *TREX2* methylation decrease were mainly derived from epithelia potentially exposed to environmental toxins, such as the skin, lung, colon, bladder, and the head and neck area. We hypothesize that TREX2 may have a common mode of regulation in these tissues in order to counteract DNA damage by environmental genotoxic agents. Reducing DNA methylation at the *TREX2* locus followed by increased expression may provide cells with a possibility of epigenetic adaptation to environmental challenges.

Moreover, we observed a weak trend for an increase of *TREX2* mRNA expression in tumor samples compared to controls in several TCGA datasets. We hypothesize that this seemingly contradictory finding highlights *TREX2* as part of a tumor suppressive transcriptional response possibly triggered by oncogenesis and consequential replication stress and DNA damage [30]. Transcription could remain active even in later stages of malignant development, with potential adaptation of cancer cells or posttranscriptional counter-regulatory mechanisms in place. In fact, the relatively weak correlation of TREX2 mRNA expression and methylation in some cancers points to additional layers of regulation on the posttranscriptional level. In the TCGA subgroup of laryngeal cancer and adjacent normal tissue samples, *TREX2* mRNA expression is not significantly different (Fig. 2d), potentially indicating that TREX2 upregulation may already take place in non-malignant tissue impacted by pre-malignant deregulation and field cancerization effects frequently observed in laryngeal tumors [31, 32]. Additionally, heterogeneity in TREX2 protein and transcript variants has been proposed before [25, 33], and our own data indicate similar heterogeneity which deserves further investigation in the future.

A further important role of *TREX2* induction has been observed in response to inflammatory stimuli [27]. Recent studies of TREX2 function have revealed its association with apoptosis induction and immune stimulation [19, 27, 34]. Of note, the immune equilibrium of the skin is critically dependent on TREX2 and its ability to process immune signals arising from immunogenic cell death and DNA breakdown [35]. We suggest that the association which we see between methylation loss and increased *TREX2* expression could be responsible for beneficial downstream events like improved immune response and the survival benefit observed in a subgroup of laryngeal cancer patients. The growing role of immune therapies in HNSCC [36] warrants further investigation of epigenetically diverse DNA repair factors like *TREX2* in immune surveillance and possibly immunotherapy response [37].

The described contribution of TREX2 protein to DNA double-strand break repair [21, 22] might highlight this protein as an interesting target for potential cancer therapies. High TREX2 expression makes cells favor the canonical non-homologous end joining (NHEJ) pathway over alternative end joining, thus inducing an increase in distinct chromosomal rearrangements contributing to tumorigenesis [38]. This might also have implications for tumor treatments targeting DNA double-strand break repair. High TREX2 expression might render cells uniquely dependent on canonical end joining, especially in the absence of ATM, and thus might open new possibilities for treatments based on synthetic lethality effects.

In summary, our findings indicate a multilayered, conserved epigenetic regulation for *TREX2*. This is supported by our molecular analysis which revealed that the *TREX2* locus affected by methylation loss has gene enhancer activity and likely drives gene expression of *TREX2 in cis* by serving as a transcription factor binding site for CEBPA and possibly other factors. We found conserved CEBPA binding sites in both the *TREX2* enhancer and promoter and showed that both regions respond to altered CEBPA levels. We propose that these two regions share a common mode of regulation by binding of the same transcription factor. CEBPA has been reported as a tumor suppressor protein in various cancers, including HNSCC [39]. Together with *TREX2* DNA methylation, the presence of CEBPA provides a second layer of gene regulation at this genomic site.

## Conclusions

Our work provides a basis for the understanding of differential *TREX2* regulation in cancer. *TREX2* levels are correlated with DNA methylation at an intragenic gene regulatory site indicative for survival in HNSCC. Remarkably, methylation variation was detected in the adjacent non-tumor tissues, suggesting that DNA methylation could already be altered in these tissues, probably due to field cancerization effects by chronic carcinogen exposure [31, 32]. Thus, we conclude that *TREX2* DNA methylation might be useful as a biomarker to understand carcinogenesis in stratified epithelia and as a possible predictor of treatment response. In particular, tumors with high TREX2 expression might be less aggressive or respond better to specific therapies exploiting DNA damage response pathways.

## Methods

### Patient samples and clinical data

Tissue samples were obtained from patients recruited in a population-based case-control study which was carried out in the Rhein-Neckar-Odenwald region, south west of Germany [7]. The study included laryngeal cancer patients treated in clinics of the cities Heidelberg, Mannheim, Ludwigshafen, Darmstadt, and Heilbronn. Ascertainment of histologically confirmed laryngeal cancer cases occurred from 1998 to 2000 for a final sample size of 248 cases (age 36 to 80 years). Socio-demographic data and information on smoking, alcohol consumption, occupational exposure, family history of cancer, and nutrition was collected at the time of recruitment with a standardized questionnaire (Table 1). At 5 and 10 years of follow-up, information on clinical information was collected from physician records. Suitable DNA samples were obtained from FFPE tissue sections of tumors from 181 study patients. Clinically normal head and neck mucosa samples from non-cancer patients who underwent tonsillectomy were obtained from the Department of Otorhinolaryngology, Head and Neck Surgery, University of Heidelberg, via the tissue bank of the NCT Tissue Bank, Heidelberg, Germany. The validation set for CRC consisted of 64 CRC tissue samples and 29 samples from adjacent normal tissues (Department of Pathology, Hong Kong University). Patients had a mean age of 57 (range 25–83 years) and included 34 (52%) females. For further clinical features, see previous work [13].

### Cell culture and reagents

Cell lines were maintained at 37 °C in a humidified 5% CO_2_ atmosphere in DMEM/10% fetal bovine serum (FBS) (Invitrogen) unless stated otherwise. HEK293T cells were purchased from ATCC. HCT116 cells were a gift from B. Vogelstein (Ludwig Center, Baltimore, MD, USA). The colon cancer cell lines CaCo2 and SW48 were obtained from J. Hoheisel (DKFZ, Heidelberg, Germany), and KM12, RKO, LS174T, and DLD1 from T. Dick (DKZF). The neoplastic lymphocyte cell lines EHEB, HH, Jurkat, Raji, and MEC1 cells were provided by M. Daskalakis (DKFZ). HNSCC cell lines [40] HNO388, HNO447, HNO97, and HNO206 were obtained from C. Herold-Mende (Department of Otorhinolaryngology, University of Heidelberg). SW48, DLD1, EHEB, HH, Jurkat, Raji, and MEC1 cells were grown in RPMI 1640/10% FBS (Invitrogen). Primary normal human epidermal keratinocytes (NHEK) were obtained and cultivated in low-calcium, serum-free DermaLife K medium (Lifeline) as described [41]. Cells were routinely tested for the absence of mycoplasma contamination using the Venor GeM kit (Minerva Biolabs). Cell line authenticity and purity were confirmed using the Multiplex Cell Authentication and Cell Contamination Test (Multiplexion). The SNP profiles matched known profiles or were unique for the HNO cell lines. No mycoplasma, SMRV, or interspecies contamination was detected. 5-Aza-dC (Sigma-Aldrich) was dissolved in PBS and used with daily media change and re-dosing.

### siRNA transfection

siRNA transfection of cell lines was carried out using INTERFERin (Polyplus transfection). Cells were transfected using 1.0 μl transfection reagent per 0.02 pmol siRNA, and all siRNAs (GE Dharmacon) were used as a pool of four individual sequences at a combined final concentration of 10 nM (Cat.-No.: D-006422-02/04/05/19 for CEBPA with target sequences CAGAGAGCUCCUUGGUCAA, ACAAGAACAGCAACGAGUA, CGGUGGACAAGAACAGCAA, and GGAACACGAAGCACGAUCA). Luciferase reporter assays were set up as described 48 h after siRNA transfection.

### Quantitative DNA methylation analysis using EpiTYPER

High-resolution DNA methylation analysis was carried out using EpiTYPER MassARRAY technology (Agena Bioscience) as described [42]. For formalin-fixed tissue sections, we utilized an adjusted DNA isolation protocol based on a commercially available isolation method (QIAGEN). In short, three 7.5-μm paraffin tissue sections were deparaffinized in xylene at 65 °C, washed with 96% ethanol, and digested overnight with proteinase K (QIAGEN), followed by RNase A treatment and isolation of DNA with QIAamp MinElute columns according to the manufacturer’s instructions (QIAGEN). Genomic DNA (1.0 μg) was bisulfite-converted using the EZ DNA methylation kit (Zymo Research), and regions of interest were amplified by PCR. Primers (Additional file 1: Table S5) were designed using EpiDesigner software (Agena). Overall, DNA molecular size is limited by fragmentation of DNA obtained from formalin-fixed tissues. We adjusted the EpiTYPER assay to allow for the analysis of short DNA fragments by limiting the amplicon size to usually below 200 bp. For generation of DNA methylation standards, we carried out in vitro whole-genome amplification of commercially available human genomic DNA (Roche) using the RepliG mini kit (QIAGEN) methylation. Whole-genome-amplified DNA was methylated in vitro using M.SssI CpG methyltransferase (Thermo Fisher Scientific). Individual methylation standard samples were prepared by mixing methylated and unmethylated genomic DNA prior to bisulfite conversion in order to represent the indicated methylation values (0, 20, 40, 60, 80, and 100% methylation). Unless stated otherwise, DNA methylation values were calculated as average methylation of all available CpG sites within each PCR product.

### Chromatin immunoprecipitation

Chromatin immunoprecipitation for histone modifications was carried out as described previously [42]. Antibodies against H3K27ac (39133, 1:100 dil., Active Motif) and H3K4me1 (ab8895, 1:100 dil., Abcam) were used. Subsequent quantification was run on a LightCycler 480 with PCR primers for Universal ProbeLibrary (Additional file 1: Table S5). Signals were normalized to non-immunoprecipitated chromatin controls (input).

### Luciferase reporter assays

Luciferase reporter assays were carried out as described previously [42]. Briefly, genomic regions of interest were amplified by PCR from dermal fibroblast genomic DNA and cloned into pGL4.10, pGL4.23 (Promega), or pCpGfree-promoter-lucia (Invivogen). Open reading frames were obtained from the Genomics and Proteomics core facility (DKZF) and cloned into pDest11-based Gateway expression vectors (Thermo Fisher Scientific). Reporter constructs and open reading frames were validated by Sanger sequencing (GATC Biotech, Constance, Germany). All cell lines were transfected with 40 ng of reporter plasmid and 5 ng of open reading frame plasmid using TransIT-LT1 transfection reagent (Mirus Bio) in 384-well plates. Readout was carried out 48 h after transfection. Data were normalized to co-transfected luciferase reporter vectors (pRL-TK-renilla luciferase (Promega) for pGL4-based reporters and pGl4-CMV-firefly luciferase for pCpGfree-lucia reporters). In vitro methylation of reporters was carried out using M.SssI CpG methyltransferase (Thermo Fisher Scientific).

### mRNA expression analysis

Total cellular RNA was isolated using TRIzol (Invitrogen,) according to standard protocols. mRNA expression was measured using complementary DNA samples generated from 1.0 μg DNase I-treated RNA with SuperScript III Reverse Transcriptase (Invitrogen) and random hexamers (QIAGEN). Complementary DNA was analyzed with a LightCycler 480 real-time PCR system (Roche) and human Universal ProbeLibrary hydrolysis probes (Roche), using LightCycler DNA Probes Master polymerase mix (Roche). Data were normalized to housekeeping gene expression values of beta actin (*ACTB*), glyceraldehyde-3-phosphate dehydrogenase (*GAPDH*), and hypoxanthine phosphoribosyltransferase 1 (*HPRT1*), and the average of the three normalized expression values was taken for individual samples. All primers (Additional file 1: Table S5) were designed using the Universal ProbeLibrary Assay Design Center application (Roche).

### Immunoenzyme staining of TREX2 protein

Human laryngeal cancer samples from the Rhein-Neckar Laryngeal Cancer Cohort were provided by the tissue bank of the National Center for Tumor Diseases (NCT; Heidelberg, Germany) in accordance with the regulations of the tissue bank and the approval of the ethics committee of Heidelberg University. Immunoenzyme staining was performed on 2-μm sections of formalin-fixed, paraffin-embedded samples in an automated fashion with the Benchmark Ultra Stainer (Ventana Medical) using the pre-treatment protocol CC1 for 40 min. The primary antibody (anti-TREX2, 1:100, Atlas Antibodies, HPA054060) was added for 24 min. Histological and immunohistochemical evaluation was carried out by a pathologist (F.L.) in a blinded fashion. Semi-quantitative evaluation of protein expression was done using the H-score method [43]. The percentage of cells at different staining intensities was determined by visual assessment, with the score calculated using the formula 1 × (% of 1+ cells) + 2 × (% of 2+ cells) + 3 × (% of 3+ cells). Samples were classified as negative (0 = H-score 0–50), weakly positive (1 = H-score 51–100), moderately positive (2 = H-score 101–200), or strongly positive (3 = H-score 201–300). The average H-score values for each tumor and each adjacent non-tumorous tissue were calculated and compared.

### Proximity ligation assay

Proximity ligation assay was carried out as published before [44], with some modifications. HEK293T cells were transfected with plasmids for overexpression of FLAG-tagged CEBPA, and nuclear lysates were collected 48 h after transfection using the NE-PER Nuclear and Cytoplasmic Extraction Reagents (Thermo Fisher Scientific). Oligonucleotide-coupled anti-FLAG antibody (Clone M2, Sigma-Aldrich) was generated using Thunderlink oligonucleotide conjugation kit (Innova Biosciences) and a 5′-amino-modified DNA oligo (Additional file 1: Table S5). Proximity ligation probes (200 pM, Additional file 1: Table S5), conjugated antibody, and equal nuclear protein amounts were incubated at room temperature in 10 mM Tris buffer (pH 7.5) with 50 mM NaCl, 1 mM DTT, 1 mM EDTA, 5% (vol/vol) glycerol, and 1 μg of poly(dI-dC) for 2 h followed by 1 h of ligation at 16 °C. Ligation efficiency was analyzed by real-time PCR using the LightCycler 480 system and human Universal ProbeLibrary hydrolysis probes (Roche).

### *TREX2* expression and DNA methylation analysis in TCGA data

Raw data (*.idat files) on DNA methylation of various tumors were obtained from publicly available TCGA data sources (Additional file 1: Table S1). For inter- and intra-sample data normalization, raw data was BMIQ-normalized using the RnBeads R-package [45] (http://rnbeads.mpi-inf.mpg.de/). For quality filtering, the single-nucleotide polymorphism (SNP)-calling probes (dbSNP132 Common, *n* = 92,428) and probes that had detection *p* values below 0.01 in at least one sample were excluded as well as probes with missing information for a single sample per cancer study. Probes measuring methylation in a non CpG context (*n* = 3156) were removed. Methylation data were corrected for tumor tissue sample purity as described [46]. Differential methylation analysis was conducted on single CpG site and region level according to the sample groups specified in the analysis.

### Statistics

Results show mean and standard deviation unless indicated otherwise. For comparisons, two-tailed Student’s *t* test or Wilcoxon’s test was used and results with *p* values < 0.05 were considered statistically significant. Linear correlation was assessed using the Pearson correlation coefficient (*R*). Data were visualized with GraphPad Prism version 7 (GraphPad Software). Gene maps are from the UCSC genome browser. Transcription factor binding sites were predicted using TRANSFAC [47], JASPAR [48], PROMO [49], and ConSite [50] with their respective default settings.

To analyze the effect of DNA methylation on cancer survival, we performed Cox proportional hazard regression and calculated hazard ratios (HRs) with 95% confidence intervals (95% CIs). DNA methylation was included in the model as a continuous variable. The time variable was days since study entry, and the end-point of the model was death; thus, surviving individuals were censored at the end of the study. In addition to univariable analysis, we also calculated HRs adjusted for age and sex, whereby age was defined as the exact age at study entry/event and was included as a continuous variable in the model. The analysis was restricted to cancer sites with at least 20 deceased individuals. Analyses were performed using SAS 9.4.

## Additional files

Additional file 1: **Figure S1.** Differential DNA methylation at the TREX2 locus in the TCGA head and neck squamous cell carcinoma cohort. **Figure S2.** Correlation of TREX2 mRNA expression and DNA methylation in the TCGA HNSC cohort. **Figure S3.** Differential DNA methylation and TREX2 expression affect overall survival of laryngeal cancer patients. **Figure S4.** Identification of the TREX2 gene promoter in luciferase reporter assays. **Figure S5.** Validation of the identified TREX2 promoter in FANTOM5 CAGE-seq data. **Figure S6.** Chromatin immunoprecipitation of H3K4me1 at the TREX2 gene locus. **Figure S7.** Correlation of TREX2 mRNA expression in different cell lines and primary cells (*n*=15) with mRNA expression of transcription factors with predicted binding motifs at the TREX2 DMR. **Figure S8.** Induction of TREX2 gene regulatory elements by CEBPB. **Figure S9.** Luciferase reporter assays for different TREX2 promoter and DMR constructs. **Figure S10.** Proximity ligation assay for predicted CEBPA binding sites at the TREX2 locus. **Table S1A.** Genome-wide datasets on transcriptional and epigenetic alterations in cancers from TCGA (https://portal.gdc.cancer.gov/) for 22 cancer types. **Table S1B.** Differential methylation of two CpG sites located in the differentially methylated TREX2 region for matched pairs of tumor and adjacent normal tissue from TCGA cancer studies. **Table S1C.** Differential methylation of two CpG sites located in the differentially methylated TREX2 region for all tumor and normal adjacent tissues with DNA methylation values from TCGA cancer studies. **Table S2A,B.** TREX2 DNA methylation in tumor tissue and overall survival in TCGA cancer studies. **Table S3.** TREX2 mRNA expression in tumor tissue (given as log2 (normalized expression + 1)) and overall survival in TCGA cancer studies. **Table S4.** Prediction of transcription factor binding sites at the TREX2 DMR. **Table S5.** DNA oligonucleotides used for DNA methylation analysis, qRT-PCR, ChIP-qPCR, proximity ligation assay, and molecular cloning. (DOCX 1615 kb)

## Abbreviations

5-aza-dC: 5-Aza-2′-deoxycytidine
CAGE: Cap analysis gene expression
CEBPA: CCAAT/enhancer binding protein alpha
CEBPB: CCAAT/enhancer binding protein beta
ChIP: Chromatin immunoprecipitation
COAD: Colon adenocarcinoma
CRC: Colorectal cancer
DMR: Differentially methylated region
FFPE: Formalin-fixed paraffin-embedded
H3K27ac: Histone H3 lysine 27 acetylation
H3K4me1: Histone H3 lysine 4 monomethylation
HNSC/HNSCC: Head and neck squamous cell carcinoma
IHC: Immunohistochemistry
LIHC: Liver hepatocellular carcinoma
LUSC: Lung squamous cell carcinoma
NHEK: Normal human epidermal keratinocytes
PCPG: Pheochromocytoma and paraganglioma
TCGA: From the Cancer Genome Atlas
TREX2: Three prime repair exonuclease 2
