# Supplementary material for: DNA methylation at an enhancer of the three prime repair exonuclease 2 gene (TREX2) is linked to gene expression and survival in laryngeal cancer
Source: Clin Epigenetics. 2019 May 3;11:67. doi: 10.1186/s13148-019-0666-5 (PMC6499986; doi:10.1186/s13148-019-0666-5)
Supplement: Supplementary file 1 — Figure S1. Differential DNA methylation at the TREX2 locus in the TCGA head and neck squamous cell carcinoma cohort. Figure S2. Correlation of TREX2 mRNA expression and DNA methylation in the TCGA HNSC cohort. Figure S3. Differential DNA methylation and TREX2 expression affect overall survival of laryngeal cancer patients. Figure S4. Identification of the TREX2 gene promoter in luciferase reporter assays. Figure S5. Validation of the identified TREX2 promoter in FANTOM5 CAGE-seq data. Figure S6. Chromatin immunoprecipitation of H3K4me1 at the TREX2 gene locus. Figure S7. Correlation of TREX2 mRNA expression in different cell lines and primary cells (n=15) with mRNA expression of transcription factors with predicted binding motifs at the TREX2 DMR. Figure S8. Induction of TREX2 gene regulatory elements by CEBPB. Figure S9. Luciferase reporter assays for different TREX2 promoter and DMR constructs. Figure S10. Proximity ligation assay for predicted CEBPA binding sites at the TREX2 locus. Table S1A. Genome-wide datasets on transcriptional and epigenetic alterations in cancers from TCGA (https://portal.gdc.cancer.gov/) for 22 cancer types. Table S1B. Differential methylation of two CpG sites located in the differentially methylated TREX2 region for matched pairs of tumor and adjacent normal tissue from TCGA cancer studies. Table S1C. Differential methylation of two CpG sites located in the differentially methylated TREX2 region for all tumor and normal adjacent tissues with DNA methylation values from TCGA cancer studies. Table S2A,B. TREX2 DNA methylation in tumor tissue and overall survival in TCGA cancer studies. Table S3. TREX2 mRNA expression in tumor tissue (given as log2 (normalized expression + 1)) and overall survival in TCGA cancer studies. Table S4. Prediction of transcription factor binding sites at the TREX2 DMR. Table S5. DNA oligonucleotides used for DNA methylation analysis, qRT-PCR, ChIP-qPCR, proximity ligation assay, and molecular cloning. (DOCX 1615 [file 13148_2019_666_MOESM1_ESM.docx]

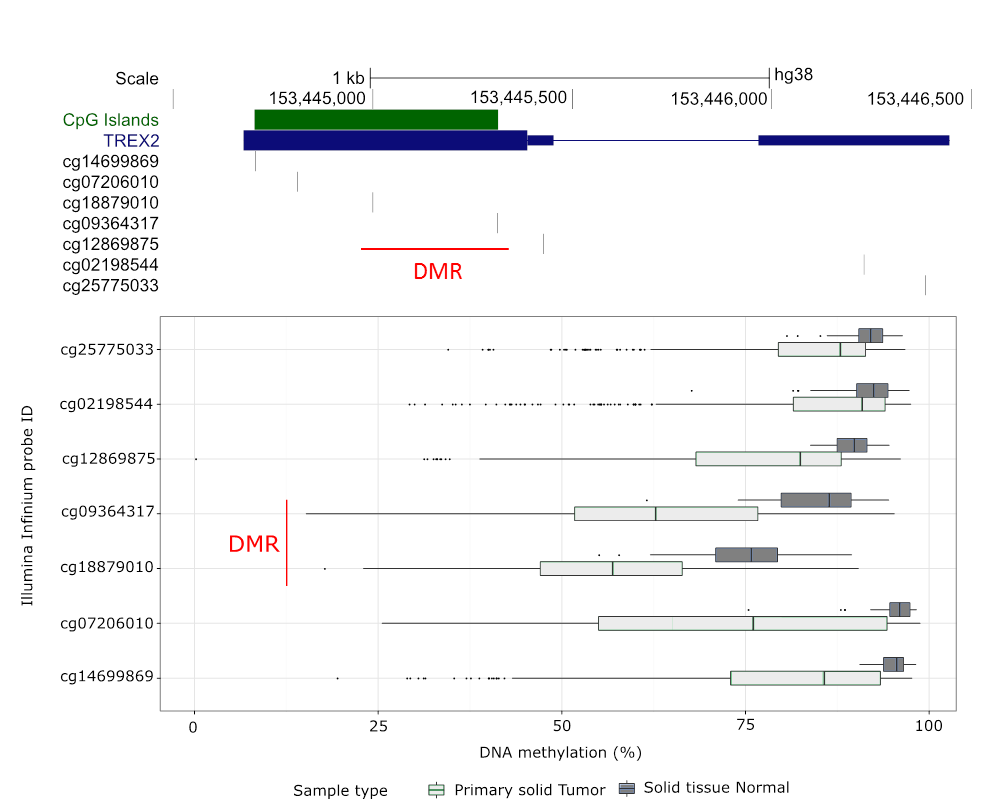


**Fig. S1.** Differential DNA methylation at the *TREX2* locus in the TCGA head and neck squamous cell carcinoma cohort. Upper panel: Map of the *TREX2* gene locus with Illumina Infinium BeadChIP CpG probe locations, *TREX2* transcript (blue) and CpG islands (green) indicated. DMR-associated CpG-probes are marked (red). lower panel: Data depict CpG methylation at Illumina Infinium HumanMethylation 450 BeadChIP probes in HNSC. Box-Whisker plot shows mean and the 10th to 90th percentiles in tumor (n=528) and adjacent non-tumor tissue (n=50) samples. Whiskers extend from the hinge to the largest or smallest value no further than 1.5 * IQR from the hinge (where IQR is the inter-quartile range or distance between the first and third quartiles). Data beyond the end of the whiskers are called "outlying" points and are plotted individually. This gives a roughly 95% confidence interval or comparing medians.

**
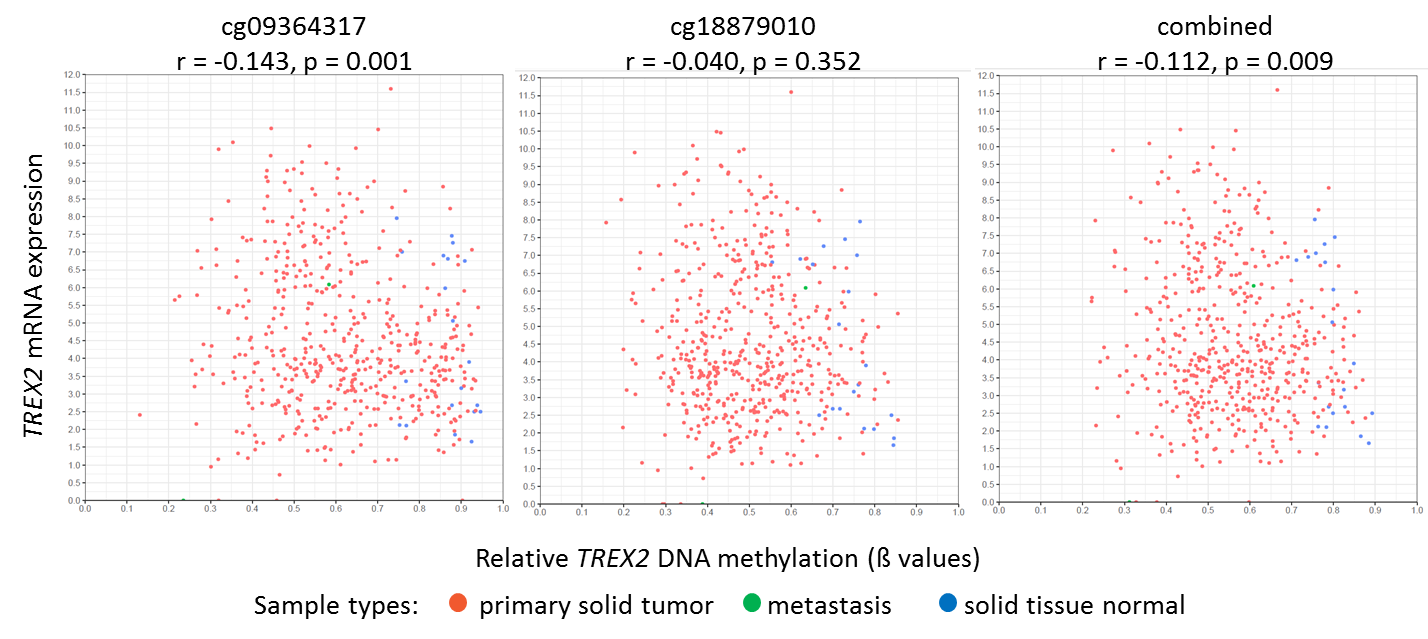
**

**Fig. S2.** Correlation of *TREX2* mRNA expression and DNA methylation in the TCGA HNSC cohort (n=542, red dots: primary solid tumor, green dots: metastatic tumor, blue dots: solid normal tissue). Data show matched *TREX2* mRNA expression from RNA-seq and *TREX2* DMR methylation levels from Illumina Infinium HumanMethylation 450 BeadChip microarrays. TREX2 expression is given as log2(x+1) transformed RSEM normalized counts and methylation as ß values for cg09364317, cg18879010 and the mean values of both. Correlation coefficient and p value for 100000 permutations are shown.

**
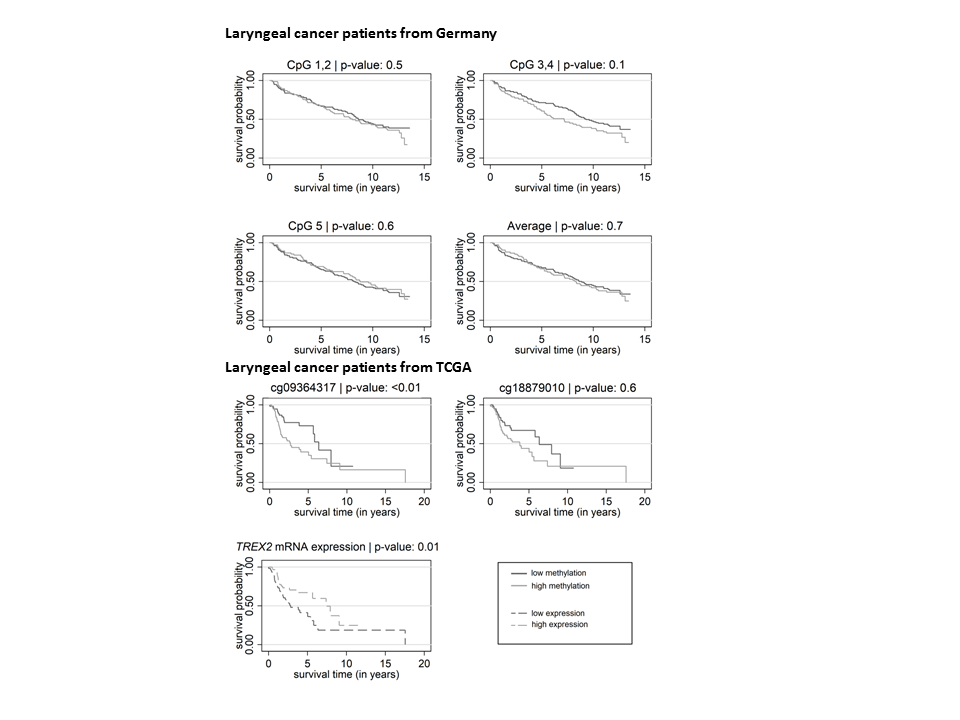
**

**Fig. S3**. Differential DNA methylation and *TREX2* expression affect overall survival of laryngeal cancer patients. Kaplan-Meier survival curves are shown for high (light grey) *versus* low (dark grey) *TREX2* DNA methylation or low expression (dark grey) versus high (light grey) *TREX2* mRNA in tumor samples from laryngeal cancer patients from Germany or TCGA. Data are based on methylation values for the EpiTYPER TREX2_2 amplicon, the most informative Illumina 450K array probes cg09364317 and cg18879010, or *TREX2* mRNA expression. Grouping of patients in sub-categories was based on the median methylation of each respective CpG site or gene expression (log2(x+1) transformed RSEM normalized counts).

**Fig. S4.** Identification of the *TREX2* gene promoter in luciferase reporter assays. (**a**) Left panel: Map of the *TREX2* gene locus with regions used for luciferase reporter assays (black), Refseq gene transcripts (blue), CpG islands (green) and sequence conservation across vertebrates (Cons 100 Verts UCSC genome browser). *TREX2* regions with promoter activity are highlighted in orange. Right panel: Heat map depicting relative luciferase signal in different cell lines transfected with *TREX2* luciferase reporter constructs, with reporter names aligned to respective regions shown in left panel. (**b**) Left panel: Map of the *TREX2* gene locus with regions used for luciferase reporter assays (black) and *TREX2* gene transcript (blue). Arrows indicate direction of reporter insert into luciferase vector (inv: inverted). Right panel: Heat map depicting relative luciferase signal in different cell lines transfected with *TREX2* luciferase reporter constructs, with reporter names aligned to respective regions shown in left panel. All luciferase signals depict mean of quadruplicates normalized to empty vector (pGl4.10) control, with values ranging from 1 (white) to 50-fold (orange).

**Fig. S5.** Validation of the identified *TREX2* promoter in FANTOM5 CAGE-seq data. Map of the *TREX2* gene locus with CAGE-seq peak (grey), promoter region identified in luciferase reporter assays (black, PROM1 and PROM2), Refseq gene transcripts (blue), CpG islands (green) and sequence conservation across vertebrates (Cons 100 Verts, UCSC genome browser).

**Fig. S6.** Chromatin immunoprecipitation of H3K4me1 at the *TREX2* gene locus. Upper panel: Map of the *TREX2* gene locus with ChIP-qPCR amplicons, regions used for luciferase reporter assays, *TREX2* transcript (blue) and CpG islands (green) indicated. Lower panel: ChIP-qPCR assays in different cell lines at the *TREX2* locus. Bars show HK4me1 signal at four regions of the *TREX2* gene (see upper panel). All ChIP assays show mean and standard deviation from four replicates. Dot plot shows correlation of H3K4me1 signal at the *TREX2* DMR (ChIP region 3) and relative *TREX2* mRNA expression determined in cell lines (n=6).

**Fig. S7.** Correlation of *TREX2* mRNA expression in different cell lines and primary cells (n=15) with mRNA expression of transcription factors with predicted binding motifs at the *TREX2* DMR. Heat map shows relative mRNA expression determined via qRT-PCR with values ranging from 0 (white) to maximum expression of the corresponding gene (orange). Mean of duplicate measurements is shown. Respective R^2^ value for linear correlation with *TREX2* is indicated below each column. CRC: Colorectal cancer; HNSCC: Head and neck squamous cell carcinoma.

**Fig. S8.** Induction of *TREX2* gene regulatory elements by CEBPB. Upper panel: Map of the *TREX2* gene locus with predicted CEBPA binding sites, regions used for luciferase reporter assays, *TREX2* transcript (blue) and CpG islands (green). Lower panel: Heat map depicting relative luciferase signal in different cell lines transfected with *TREX2* luciferase reporter constructs. Schematic view of reporters is shown to the left, with reporter names referring to *TREX2* regions shown in Fig. 4D. The promoter-upstream region TREX2_4 was included as a negative control. Luciferase signals depict mean of duplicates normalized to empty vector (pGl4.23) control, with values ranging from 1 (white) to 100-fold (orange). Co-transfections of CEBPA overexpression plasmid (CEBPA), the negative regulator isoform CEBPB liver inhibitory protein (LIP), or the fully functional CEBPB liver activating protein (LAP) isoform are indicated. 6X CEBPA: synthetic CEBPA pathway reporter element with 6 tandem CEBPA consensus binding sites. minP/Luc2: minimal promoter/luc2 luciferase gene included in the pGl4 vector.

**Fig. S9.** (**a-d**) Luciferase reporter assays for different *TREX2* promoter and DMR constructs (for location of sequences see Fig. 4D, upper panel) in cell lines under co-treatment with siRNA directed against *CEBPA (*siCEBPA*)*. Schematic view of the transfected reporter construct (*TREX2* promoter and DMR) is included. (**e,f**) Assessment of *CEBPA* knockdown via siRNA and effect on endogenous *TREX2* transcript in cell lines. Data show relative cellular CEBPA (e) and *TREX2* (f) mRNA expression 96h after siRNA pool transfection. Bars depict mean and standard deviation from quadruplicate experiments. Luc2: luc2 luciferase gene included in the pGl4 vector.

**Fig. S10.** Proximity ligation assay for predicted CEBPA binding sites at the *TREX2* locus. Upper panel: Map of the *TREX2* gene locus with CEBPA binding sites tested in proximity ligation assay, *TREX2* transcript (blue) and CpG islands (green). Lower panel: Bar chart with relative ligation efficiencies of CEBPA oligonucleotides incubated with protein extract from CEBPA-overexpressing HEK293T cells (CEBPA) versus Empty vector-transfected cell extracts. Mean and standard deviation of quadruplicate experiments is shown. Binding motifs (bold) and respective ligated oligonucleotide sequences are depicted to the left

**Table S1A.** Genome-wide datasets on transcriptional and epigenetic alterations in cancers from TCGA (https://portal.gdc.cancer.gov/) for 22 cancer types, covering 7018 tumor samples used for the analysis of differential methylation (Tables S2B and C), and for differential expression and survival analysis TCGA PANCAN data sets consist of methylation data (Illumina Infinium HumanMethylation 450 microarrays). Numbers (tumor and normal tissue) refer to samples with matched transcriptome/methylome datasets available and include samples with matched adjacent normal tissue data.

|  |  | **DNA methylation** | | | **Gene expression** | | | | |  |
| --- | --- | --- | --- | --- | --- | --- | --- | --- | --- | --- |
| **TCGA study code** | **Cancer type** | **Tumor** | **Normal** | **Matched pairs** | **Tumor** | **Normal** | **Matched pairs** | |  |  |
|  |  | **N** | **N** | **N** | **N** | **N** | | **N** | | |
| **BLCA** | Bladder urothelial carcinoma | 409 | 21 | 20 | 408 | 19 | | 19 | | |
| **BRCA** | Breast invasive carcinoma | 777 | 97 | 90 | 1095 | 113 | | 113 | | |
| **CESC** | Cervical squamous cell carcinoma and endocervical adenocarcinoma | 307 | 3 | 3 | 304 | 3 | | 3 | | |
| **CHOL** | Cholangiocarcinoma | 36 | 9 | 9 | 36 | 9 | | 9 | | |
| **COAD** | Colon adenocarcinoma | 263 | 38 | 37 | 285 | 41 | | 41 | | |
| **GBM** | Glioblastoma multiforme | 140 | 2 | 1 | 152 | 5 | | 5 | | |
| **HNSC** | Head and neck squamous cell carcinoma | 528 | 50 | 50 | 520 | 44 | | 44 | | |
| **KIRC** | Kidney renal clear cell carcinoma | 316 | 160 | 160 | 533 | 72 | | 72 | | |
| **KIRP** | Kidney renal papillary cell carcinoma | 275 | 45 | 45 | 290 | 32 | | 32 | | |
| **LIHC** | Liver hepatocellular carcinoma | 377 | 50 | 50 | 371 | 50 | | 50 | | |
| **LUAD** | Lung adenocarcinoma | 447 | 32 | 23 | 515 | 59 | | 59 | | |
| **LUSC** | Lung squamous cell carcinoma | 370 | 42 | 40 | 500 | 51 | | 51 | | |
| **PAAD** | Pancreatic adenocarcinoma | 184 | 10 | 10 | 178 | 4 | | 4 | | |
| **PCPG** | Pheochromocytoma and paraganglioma | 179 | 3 | 3 | 179 | 3 | | 3 | | |
| **PRAD** | Prostate adenocarcinoma | 494 | 50 | 50 | 497 | 52 | | 52 | | |
| **READ** | Rectal adenocarcinoma | 98 | 7 | 0 | 94 | 10 | | 10 | | |
| **SARC** | Sarcoma | 261 | 4 | 4 | 21 | 2 | | 2 | | |
| **SKCM** | Skin Cutaneous Melanoma | 104 | 2 | 0 | 103 | 1 | | 1 | | |
| **STAD** | Stomach adenocarcinoma | 395 | 2 | 2 | 415 | 35 | | 35 | | |
| **THCA** | Thyroid carcinoma | 507 | 56 | 56 | 505 | 59 | | 59 | | |
| **THYM** | Thymoma | 124 | 2 | 2 | 120 | 2 | | 2 | | |
| **UCEC** | Uterine Corpus Endometrial Carcinoma | 427 | 46 | 33 | 176 | 24 | | 24 | | |
|  |  |  |  |  |  |  | |  | | |

**Table S1B.** Differential methylation of two CpG sites located in the differentially methylated TREX2 region for **matched pairs** of tumor and adjacent normal tissue from TCGA cancer studies. TCGA PANCAN data sets with methylation data from Illumina Infinium HumanMethylation 450 microarrays were used.

| **TCGA study** | **DNA methylation difference** | | | | | | | | |
| --- | --- | --- | --- | --- | --- | --- | --- | --- | --- |
|  | **cg18879010** | | | **cg09364317** | | | **cg18879010 and cg09364317** | | |
|  | **percent^1^** | **P value^2^** | P value ^3^ | **percent^1^** | **P value^2^** | P value ^3^ | **percent^1^** | **P value^2^** | P value ^3^ |
| BLCA | -31.7 | 3.81E-06 | **1.60E-05** | -20.3 | 2.67E-05 | **0.0001** | -26.0 | 3.81E-06 | **1.60E-05** |
| BRCA | 1.8 | 0.0209 | **0.0488** | -4.7 | 0.0047 | **0.0098** | -1.4 | 0.6207 | 0.6860 |
| CESC | -18.2 | 0.5000 | 0.5526 | -17.4 | 0.2500 | 0.4038 | -17.8 | 0.2500 | 0.3750 |
| CHOL | -7.9 | 0.3594 | 0.4717 | -3.4 | 0.7344 | 0.8117 | -5.6 | 0.4961 | 0.5833 |
| COAD | -23.7 | 2.15E-07 | **1.50E-06** | -19.7 | 3.36E-06 | **1.77E-05** | -21.7 | 1.89E-07 | **9.90E-07** |
| GBM | -10.4 | 1.0000 | 1.0000 | -5.1 | 1.0000 | 1.0000 | -7.8 | 1.0000 | 1.0000 |
| HNSC | -26.1 | 1.51E-09 | **1.59E-08** | -24.3 | 6.20E-09 | **1.30E-07** | -25.2 | 1.61E-09 | **1.69E-08** |
| KIRC | -3.0 | 0.0346 | 0.0726 | -4.7 | 0.0005 | **0.0013** | -3.8 | 1.08E-05 | **3.79E-05** |
| KIRP | -0.1 | 0.1597 | 0.2803 | -0.3 | 0.3700 | 0.5181 | -0.2 | 0.4397 | 0.5833 |
| LIHC | -10.7 | 4.23E-07 | **2.22E-06** | -20.1 | 2.04E-08 | **2.14E-07** | -15.4 | 9.26E-09 | **6.48E-08** |
| LUAD | -10.3 | 0.0039 | **0.0102** | -8.4 | 0.0277 | 0.0528 | -9.4 | 0.0043 | **0.0101** |
| LUSC | -20.3 | 1.60E-10 | **3.36E-09** | -10.8 | 0.0000 | **0.0000** | -15.6 | 5.58E-10 | **1.17E-08** |
| PAAD | -6.3 | 0.1602 | 0.2803 | -7.5 | 0.2754 | 0.4131 | -6.9 | 0.1934 | 0.3691 |
| PCPG | -26.3 | 0.2500 | 0.4038 | -43.4 | 0.2500 | 0.4038 | -34.8 | 0.2500 | 0.3750 |
| PRAD | 0.9 | 0.2972 | 0.4457 | -6.5 | 0.0003 | **0.0009** | -2.8 | 0.1008 | 0.2116 |
| SARC | -4.5 | 0.8750 | 0.9188 | -4.2 | 1.0000 | 1.0000 | -4.4 | 1.0000 | 1.0000 |
| STAD | -41.6 | 0.5000 | 0.5526 | -36.6 | 0.5000 | 0.5833 | -39.1 | 0.5000 | 0.5833 |
| THCA | -1.2 | 0.3216 | 0.4503 | -3.2 | 0.4264 | 0.5597 | -2.2 | 0.2451 | 0.3750 |
| THYM | 7.0 | 0.5000 | 0.5526 | 9.8 | 0.5000 | 0.5833 | 8.4 | 0.5000 | 0.5833 |
| UCEC | -11.0 | 2.48E-05 | **8.68E-05** | -9.1 | 0.0040 | **0.0094** | -10.0 | **0.0005** | **0.0014** |

^1^average methylation at indicated Illumina Infinium CpG probe, normal tissue minus mean of tumor tissue, ^2^Wilcoxon matched pairs signed rank test, ^3^Wilcoxon matched pairs signed rank test corrected for false discovery rate according to Benjamini-Hochberg, P values <0.05 are given in bold

**Table S1C.** Differential methylation of two CpG sites located in the differentially methylated TREX2 region for **all** tumor and normal adjacent tissues with DNA methylation values from TCGA cancer studies. TCGA PANCAN data sets with methylation data from Illumina Infinium HumanMethylation 450 microarrays were used.

| **TCGA study** | **DNA methylation difference** | | | | | | | | | |  |  |
| --- | --- | --- | --- | --- | --- | --- | --- | --- | --- | --- | --- | --- |
|  | **cg18879010** | | | **cg09364317** | | | **cg18879010 and cg09364317** | | | | |  |
|  | **percent^1^** | **P value^2^** | P value ^3^ | **percent^1^** | **P value^2^** | P value ^3^ | | **percent^1^** | **P value^2^** | P value ^3^ | | |
| BLCA | -34.1 | 1.07E-12 | 4.73E-12 | -16.2 | 1.65E-04 | 4.54E-04 | | -25.1 | 2.72E-10 | **1.20E-09** | | |
| BRCA | -1.6 | 6.56E-01 | 7.22E-01 | -6.2 | 4.27E-09 | 1.57E-08 | | -3.9 | 9.84E-05 | **2.16E-04** | | |
| CESC | -22.0 | 1.08E-02 | 2.37E-02 | -19.4 | 2.35E-02 | 3.69E-02 | | -20.7 | 9.81E-03 | **1.66E-02** | | |
| CHOL | -5.0 | 2.93E-01 | 3.39E-01 | -1.6 | 9.44E-01 | 9.44E-01 | | -3.3 | 5.48E-01 | 5.48E-01 | | |
| COAD | -21.0 | 1.55E-14 | 8.50E-14 | -19.4 | 1.51E-13 | 1.66E-12 | | -20.2 | 3.51E-16 | **1.93E-15** | | |
| GBM | -30.3 | 2.28E-02 | 3.86E-02 | -19.4 | 1.69E-01 | 1.86E-01 | | -24.8 | 2.50E-02 | **3.92E-02** | | |
| HNSC | -25.4 | 9.15E-26 | 2.01E-24 | -23.6 | 6.15E-20 | 1.35E-18 | | -24.5 | 1.03E-25 | **2.27E-24** | | |
| KIRC | -2.9 | 5.90E-03 | 1.44E-02 | -4.9 | 2.21E-02 | 3.69E-02 | | -3.9 | 7.89E-05 | **1.93E-04** | | |
| KIRP | -2.8 | 8.95E-01 | 9.38E-01 | -0.8 | 2.48E-03 | 4.96E-03 | | -1.8 | 1.29E-01 | 1.42E-01 | | |
| LIHC | -16.1 | 9.35E-18 | 6.86E-17 | -22.2 | 1.71E-12 | 1.26E-11 | | -19.2 | 3.59E-17 | **2.63E-16** | | |
| LUAD | -8.6 | 4.33E-06 | 1.36E-05 | -8.9 | 2.97E-04 | 6.54E-04 | | -8.8 | 4.31E-06 | **1.35E-05** | | |
| LUSC | -23.1 | 5.95E-23 | 6.54E-22 | -14.0 | 6.69E-11 | 3.68E-10 | | -18.5 | 1.54E-20 | **1.70E-19** | | |
| PAAD | -6.6 | 7.48E-02 | 9.14E-02 | -8.0 | 1.05E-01 | 1.21E-01 | | -7.3 | 4.76E-02 | 5.82E-02 | | |
| PCPG | -15.4 | 2.22E-02 | 3.86E-02 | -37.7 | 8.00E-03 | 1.47E-02 | | -26.5 | 6.78E-03 | **1.24E-02** | | |
| PRAD | -1.1 | 9.77E-01 | 9.77E-01 | -6.4 | 7.99E-10 | 3.52E-09 | | -3.7 | 8.70E-04 | **1.74E-03** | | |
| SARC | -15.0 | 4.32E-02 | 6.34E-02 | -19.3 | 9.30E-02 | 1.14E-01 | | -17.2 | 4.75E-02 | 5.82E-02 | | |
| SKCM | -26.9 | 5.54E-02 | 7.62E-02 | -21.9 | 3.99E-02 | 5.85E-02 | | -24.4 | 4.71E-02 | 5.82E-02 | | |
| STAD | -32.8 | 1.87E-02 | 3.75E-02 | -23.8 | 6.52E-02 | 8.43E-02 | | -28.3 | 3.18E-02 | **4.67E-02** | | |
| THCA | 0.0 | 3.36E-02 | 5.28E-02 | -3.1 | 7.46E-01 | 7.81E-01 | | -1.6 | 4.83E-01 | 5.06E-01 | | |
| THYM | 5.9 | 6.23E-02 | 8.06E-02 | 13.9 | 6.51E-02 | 8.43E-02 | | 9.9 | 6.51E-02 | 7.54E-02 | | |
| UCEC | -9.3 | 3.58E-07 | 1.31E-06 | -6.4 | 2.15E-04 | 5.26E-04 | | -7.8 | 4.26E-06 | **1.35E-05** | | |

^1^average methylation at indicated Illumina Infinium CpG probe, normal tissue minus mean of tumor tissue, ^2^Wilcoxon test, ^3^Wilcoxon test corrected for false discovery rate according to Benjamini-Hochberg, P values <0.05 are given in bold.

**Table S2A.** *TREX2* DNA methylation in tumor tissue and overall survival in TCGA cancer studies. Hazard ratios (HR) for continuous change of methylation at the Illumina 450K methylation array probe **cg09364317** are given after univariate analysis and analysis adjusted for age and gender. Cancer studies with less than 20 events were not included in the analysis, HRs with P values <0.05 are given in bold.

|  | **Univariate analysis** | | | | | | **Analysis adjusted for age and gender** | | | | | |
| --- | --- | --- | --- | --- | --- | --- | --- | --- | --- | --- | --- | --- |
| **Cancer site** | **Observations  (N)** | **Events (N)** | **Hazard  Ratio** | **95% Confidence Interval** | | **P value** | **Observations  (N)** | **Events (N)** | **Hazard Ratio** | **95% Confidence Interval** | | **P value** |
| ACC | 80 | 29 | 0.13 | 0.01 | 1.57 | 0.1081 | 80 | 29 | 0.09 | 0.01 | 1.42 | 0.0878 |
| BRCA | 775 | 103 | 0.59 | 0.12 | 2.94 | 0.5235 | 762 | 103 | 0.57 | 0.11 | 2.86 | 0.4918 |
| CESC | 307 | 71 | 0.65 | 0.15 | 2.78 | 0.5604 | 305 | 71 | 0.58 | 0.14 | 2.50 | 0.4653 |
| COAD | 261 | 64 | **0.25** | **0.07** | **0.96** | **0.0440** | 260 | 64 | 0.34 | 0.09 | 1.29 | 0.1132 |
| GBM | 138 | 93 | 0.75 | 0.27 | 2.10 | 0.5854 | 138 | 93 | 0.95 | 0.21 | 4.39 | 0.9495 |
| HNSC | 527 | 224 | 0.85 | 0.38 | 1.89 | 0.6877 | 527 | 224 | 0.69 | 0.30 | 1.60 | 0.3904 |
| KIRC | 316 | 105 | 3.50 | 0.91 | 13.50 | 0.0685 | 316 | 105 | 3.69 | 0.90 | 15.08 | 0.0691 |
| KIRP | 274 | 40 | **8.96** | **1.22** | **65.96** | **0.0314** | 270 | 40 | 9.73 | 0.85 | 111.4 | 0.0674 |
| LIHC | 376 | 132 | 0.98 | 0.46 | 2.10 | 0.9619 | 373 | 130 | 0.80 | 0.35 | 1.84 | 0.5997 |
| LUAD | 438 | 158 | 1.94 | 0.64 | 5.88 | 0.2443 | 418 | 150 | 2.36 | 0.70 | 7.94 | 0.1656 |
| LUSC | 363 | 155 | 0.66 | 0.23 | 1.88 | 0.4350 | 357 | 155 | 0.69 | 0.24 | 2.00 | 0.4939 |
| MESO | 86 | 73 | 1.92 | 0.40 | 9.32 | 0.4170 | 86 | 73 | 1.51 | 0.30 | 7.72 | 0.6217 |
| PAAD | 184 | 99 | 0.66 | 0.19 | 2.29 | 0.5072 | 184 | 99 | 0.51 | 0.13 | 1.93 | 0.3203 |
| SARC | 261 | 99 | 1.48 | 0.62 | 3.52 | 0.3811 | 260 | 98 | 1.39 | 0.57 | 3.43 | 0.4694 |
| SKCM | 104 | 30 | 1.39 | 0.14 | 13.98 | 0.7784 | 104 | 30 | 4.58 | 0.25 | 83.49 | 0.3041 |
| STAD | 391 | 151 | 1.58 | 0.65 | 3.84 | 0.3094 | 385 | 150 | 1.70 | 0.70 | 4.12 | 0.2375 |
| UCEC | 426 | 72 | 0.53 | 0.09 | 3.01 | 0.4742 | 423 | 72 | 0.87 | 0.15 | 4.86 | 0.8701 |
| UCS | 57 | 35 | 0.44 | 0.06 | 3.34 | 0.4244 | 57 | 35 | 0.62 | 0.08 | 4.73 | 0.6439 |
| UVM | 80 | 23 | 1.17 | 0.09 | 15.71 | 0.9037 | 80 | 23 | 3.65 | 0.08 | 162.7 | 0.5045 |

**Table S2B.** *TREX2* DNA methylation in tumor tissue and overall survival in TCGA cancer studies. Hazard ratios (HR) for continuous change of methylation at the Illumina 450K methylation array probe **cg18879010** are given after univariate analysis and analysis adjusted for age and gender. Cancer studies with less than 20 events were not included in the analysis. HRs with P values <0.05 are given in bold.

|  | **Univariate analysis** | | | | | | **Analysis adjusted age and gender** | | | | | | | |  |  |
| --- | --- | --- | --- | --- | --- | --- | --- | --- | --- | --- | --- | --- | --- | --- | --- | --- |
| **Cancer site** | **Observations  (N)** | **Events (N)** | **Hazard  Ratio** | **95% Confidence Interval** | | **P value** | **Observations  (N)** | **Events (N)** | **Hazard Ratio** | | **95% Confidence Interval** | | | **P value** | |  |
| ACC | 80 | 29 | 1.08 | 0.05 | 24.11 | 0.9622 | 80 | 29 | 1.01 | 0.03 | | 33.69 | 0.9964 | | | |
| BRCA | 775 | 103 | 2.09 | 0.34 | 12.76 | 0.4251 | 762 | 103 | 2.48 | 0.42 | | 14.53 | 0.3151 | | | |
| CESC | 307 | 71 | 0.52 | 0.11 | 2.39 | 0.4022 | 305 | 71 | 0.44 | 0.10 | | 2.06 | 0.2973 | | | |
| COAD | 261 | 64 | 1.31 | 0.28 | 6.14 | 0.7333 | 260 | 64 | 0.86 | 0.18 | | 4.02 | 0.8480 | | | |
| GBM | 138 | 93 | 2.46 | 0.59 | 10.31 | 0.2183 | 138 | 93 | 0.93 | 0.21 | | 4.22 | 0.9290 | | | |
| HNSC | 527 | 224 | **0.27** | **0.10** | **0.77** | **0.0140** | 527 | 224 | 0.35 | 0.12 | | 1.02 | 0.0550 | | | |
| KIRC | 316 | 105 | 1.35 | 0.15 | 12.04 | 0.7895 | 316 | 105 | 0.93 | 0.10 | | 8.41 | 0.9502 | | | |
| KIRP | 274 | 40 | **10.26** | **1.09** | **97.06** | **0.0422** | 270 | 40 | **16.62** | **1.31** | | **210.89** | **0.0302** | | | |
| LGG | 514 | 125 | **45.09** | **16.33** | **124.53** | **<.0001** | 513 | 125 | **22.31** | **8.03** | | **61.98** | **<.0001** | | | |
| LIHC | 376 | 132 | 1.13 | 0.41 | 3.11 | 0.8162 | 373 | 130 | 1.02 | 0.35 | | 2.94 | 0.9724 | | | |
| LUAD | 438 | 158 | **1.38** | **0.38** | **5.04** | **0.6302** | 418 | 150 | 1.85 | 0.51 | | 6.74 | 0.3540 | | | |
| LUSC | 363 | 155 | 0.50 | 0.17 | 1.49 | 0.2139 | 357 | 155 | 0.50 | 0.17 | | 1.53 | 0.2260 | | | |
| MESO | 86 | 73 | 7.02 | 0.36 | 137.47 | 0.1993 | 86 | 73 | 3.82 | 0.15 | | 98.33 | 0.4182 | | | |
| PAAD | 184 | 99 | 0.38 | 0.07 | 1.96 | 0.2460 | 184 | 99 | 0.34 | 0.06 | | 1.83 | 0.2090 | | | |
| SARC | 261 | 99 | **3.08** | **1.06** | **8.97** | **0.0392** | 260 | 98 | **3.58** | **1.22** | | **10.52** | **0.0206** | | | |
| SKCM | 104 | 30 | 0.45 | 0.05 | 3.81 | 0.4640 | 104 | 30 | 0.54 | 0.06 | | 4.78 | 0.5791 | | | |
| STAD | 391 | 151 | 1.92 | 0.74 | 4.99 | 0.1819 | 385 | 150 | 1.58 | 0.60 | | 4.13 | 0.3515 | | | |
| UCEC | 426 | 72 | 0.33 | 0.05 | 2.43 | 0.2788 | 423 | 72 | 0.52 | 0.07 | | 3.71 | 0.5180 | | | |
| UCS | 57 | 35 | **1.09** | **0.15** | **8.07** | **0.9347** | 57 | 35 | 1.20 | 0.17 | | 8.30 | 0.8548 | | | |
| UVM | 80 | 23 | **212.41** | **16.91** | **2667.9** | **<.0001** | 80 | 23 | **162.53** | **13.65** | | **1934.73** | **<.0001** | | | |

**Table S3.** *TREX2* mRNA expression in tumor tissue (given as log2 (normalized expression + 1)) and overall survival in TCGA cancer studies. Hazard rations (HR) and 95% confidence intervals for continuous change of gene expression are given after univariate analysis and analysis adjusted for age and gender. Cancer studies with less than 20 events were not included in the analysis, HRs with P values <0.05 are given in bold.

|  | **Univariate analysis** | | | | | | **Analysis adjusted for age and gender** | | | | | |
| --- | --- | --- | --- | --- | --- | --- | --- | --- | --- | --- | --- | --- |
| **Cancer-site** | **Observations  (N)** | **Events (N)** | **Hazard  Ratio** | **95% Confidence Interval** | | **P value** | **Observations  (N)** | **Events (N)** | **Hazard Ratio** | **95% Confidence Interval** | | **P value** |
| ACC | 79 | 28 | 1.38 | 0.95 | 2.00 | 0.0929 | 79 | 28 | 1.37 | 0.94 | 2.00 | 0.0979 |
| BRCA | 1093 | 152 | 0.96 | 0.84 | 1.10 | 0.5744 | 1093 | 152 | 1.00 | 0.87 | 1.14 | 0.9803 |
| CESC | 304 | 71 | 0.85 | 0.72 | 1.01 | 0.0592 | 304 | 71 | **0.84** | **0.72** | **1.00** | **0.0474** |
| COAD | 283 | 69 | **1.30** | **1.04** | **1.63** | **0.0204** | 283 | 69 | **1.31** | **1.04** | **1.64** | **0.0196** |
| GBM | 151 | 119 | 1.03 | 0.86 | 1.23 | 0.7388 | 151 | 119 | 1.01 | 0.84 | 1.22 | 0.9300 |
| HNSC | 519 | 221 | 0.96 | 0.90 | 1.02 | 0.1983 | 519 | 221 | 0.94 | 0.88 | 1.00 | 0.0673 |
| KIRC | 533 | 175 | 1.01 | 0.93 | 1.10 | 0.7696 | 533 | 175 | 1.03 | 0.94 | 1.14 | 0.5384 |
| KIRP | 289 | 44 | 0.98 | 0.82 | 1.18 | 0.8397 | 289 | 44 | 1.06 | 0.86 | 1.31 | 0.5628 |
| LGG | 514 | 125 | **1.50** | **1.23** | **1.82** | **<.0001** | 514 | 125 | **1.34** | **1.10** | **1.63** | **0.0037** |
| LIHC | 370 | 130 | 0.87 | 0.75 | 1.00 | 0.0548 | 370 | 130 | **0.86** | **0.75** | **1.00** | **0.0473** |
| LUAD | 506 | 183 | 1.00 | 0.88 | 1.15 | 0.9608 | 506 | 183 | 1.00 | 0.87 | 1.15 | 0.9725 |
| LUSC | 494 | 212 | 1.00 | 0.89 | 1.14 | 0.9639 | 494 | 212 | 1.00 | 0.89 | 1.14 | 0.9500 |
| MESO | 86 | 73 | 1.05 | 0.87 | 1.27 | 0.6163 | 86 | 73 | 1.07 | 0.89 | 1.29 | 0.4821 |
| PAAD | 178 | 93 | 0.99 | 0.81 | 1.21 | 0.8992 | 178 | 93 | 0.99 | 0.81 | 1.21 | 0.9139 |
| SKCM | 103 | 29 | 1.11 | 0.96 | 1.29 | 0.1433 | 103 | 29 | 1.13 | 0.97 | 1.31 | 0.1138 |
| STAD | 410 | 159 | 1.01 | 0.90 | 1.14 | 0.8516 | 410 | 159 | 0.99 | 0.88 | 1.12 | 0.9168 |
| UCEC | 175 | 32 | 1.03 | 0.79 | 1.36 | 0.8139 | 175 | 32 | 1.06 | 0.80 | 1.41 | 0.6806 |
| UCS | 57 | 35 | 1.10 | 0.78 | 1.55 | 0.6075 | 57 | 35 | 1.07 | 0.76 | 1.50 | 0.7207 |
| UVM | 80 | 23 | **0.47** | **0.34** | **0.66** | **<.0001** | 80 | 23 | **0.45** | **0.30** | **0.67** | **<.0001** |

**Table S4**. Prediction of transcription factor binding sites at the *TREX2* DMR and gene promoter (DMR and PROM2 sequences, see Fig. 4D, upper panel) using different prediction algorithms.

| **TREX2 promoter region** | | | | |
| --- | --- | --- | --- | --- |
| Sequence motif | PROMO | ConSite | TRANSFAC | JASPAR |
| USF1/2 | x | x | x |  |
| CEBPA/B | x |  | x |  |
| NF-1 | x |  | x |  |
| SP-1 | x |  | x |  |
| SOX5^1^ |  | x | x | x |
| MyoD^1^ | x |  | x | x |
| **TREX2 DMR region** | | | |  |
| Sequence motif | PROMO | ConSite | TRANSFAC | JASPAR |
| USF1/2 | x | x | x |  |
| CEBPA/B | x |  | x |  |
| NRF2 |  | x | x |  |
| c-ETS | x |  | x |  |
| ELK1 | x |  | x | x |
| AHR/ARNT | x | x |  | x |

^1^Lack of expression in HNSC TCGA cohort (RNA-seq), excluded from further analysi

**Table S5.** DNA oligonucleotides used for DNA methylation analysis, qRT-PCR, ChIP-qPCR, proximity ligation assay, and molecular cloning. UPL: LightCycler 480 Universal ProbeLibrary,

| Name  (UPL hydrolysis probe) | Forward primer sequence | | Reverse primer sequence | Coordinate (hg38) |  |
| --- | --- | --- | --- | --- | --- |
| EpiTYPER primers for methylation analysis^1^ | | | | |  |
| TREX2_1   \| TREX2_1 CpG 1 \| \| --- \| \| TREX2_1 CpG 2.3 \| \| TREX2_1 CpG 4.5 \| \| TREX2_1 CpG 6 \| \| TREX2_1 CpG 7.8 \| \| TREX2_1 CpG 9 \| \| TREX2_1 CpG 10 \| | AATTAAAGTTATTGTGGGTTATAAGGT | | CCCCTTCACTACCAAAACCAA | chrX:153445067-153445226  chrX:153445112  chrX:153445132 + 153445136  chrX: 153445143 + 153445146  chrX:153445166  chrX:153445172 + 153445174  chrX:153445197  chrX:153445206 |  |
| TREX2_2   \| TREX2_2 CpG 1.2 \| \| --- \| \| TREX2_2 CpG 3.4 \| \| TREX2_2 CpG 5 \| | GGTTAGGGGGAGGATGGTATATAGT | | CACTAAAAAACCCAATAACTTCCAA | chrX:153445364-153445547  chrX:153445512 + 15344551  chrX:153445471 + 153445475  chrX:153445425 |  |
| RT-qPCR primers | | | | |  |
| TREX2 (#21) | AAGTAGCGGTGGAAGAGGCT | | GAAGATCAGGAGCAGGGTGT | |  |
| NFIA (#20) | TCGATTTATATTTGGCATACTTTGTG | | GGTCCTTAATGTCAGCGTCAC | |  |
| NFIA (#65) | AGTCCAAGCCACAATGATCC | | TCCAGATTTTACAAAACTATCCTCAA | |  |
| NFIC (#56) | GACAGGGATGGGCTCTGAC | | TCTCCTGGAAGTCGGTCGT | |  |
| USF1 (#69) | GTGATGATGTCACCACAAGAAGTA | | CCGGGGAGCTTCTGACTT | |  |
| USF2 (#19) | CCGTACAGACCACAGACCAG | | CCTGTCTGAAGCACATCCTG | |  |
| CEBPA (#28) | GGAGCTGAGATCCCGACA | | TTCTAAGGACAGGCGTGGAG | |  |
| SP1 (#9) | CTATAGCAAATGCCCCAGGT | | TCCACCTGCTGTGTCATCAT | |  |
| ACTB (#11) | ATTGGCAATGAGCGGTTC | | GGATGCCACAGGACTCCAT | |  |
| GAPDH (#60) | GCCCAATACGACCAAATCC | | AGCCACATCGCTCAGACAC | |  |
| HPRT1 (#73) | TGACCTTGATTTATTTTGCATACC | | CGAGCAAGACGTTCAGTCCT | |  |
| Primers for ChIP-qPCR | | | | |  |
| TREX2_CHIP_2 (#69) | GCCCCGTGTTTACAAATTAGG | | GGTGGAGGTTACTGCCTTCC | chrX:153446203-153446293 |  |
| TREX2_CHIP_3 (#4) | CAAAGCTCTGACCCTTCTGG | | AGGTCACAGGTGCTGTGGT | chrX:153445547-153445607 |  |
| TREX2_CHIP_4 (#21) | GAAGATCAGGAGCAGGGTGT | | AGCCTCTTCCACCGCTACTT | chrX:153444828-153444911 | |
| TREX2_CHIP_1 (#74) | AAAGGAGATGGTGCCAGGA | | CTCCTTGGGGCCATTGTA | chrX:153447129-153447194 |  |
| DNA oligonucleotides for proximity ligation assay | | | | |  |
| CEBPA_consensus motif | ATACTATTGCACAATTGATTGCACTACATCGCCCTTGGACTACGACTGACGAACCGCTTTGCCTGACTGATCGCTAAATCTGG | | | |  |
| CEBPA_DMR1 | ATAATCAAAGCCATTGTGGGCCACAAGCATCGCCCTTGGACTACGACTGACGAACCGCTTTGCCTGACTGATCGCTAAATCTGG | | | |  |
| CEBPA_DMR2 | AGGACCCGGGGCAATACTAGGGCACATCGCCCTTGGACTACGACTGACGAACCGCTTTGCCTGACTGATCGCTAAATCTGG | | | |  |
| Connector oligo | TACTTAGACTTCCCCAGATTTAGTTT | | | |  |
| Antibody-conjugated oligo | TCGTGTCTAAAGTCCGTTACCTTGATTCCCCTAACCCTCTTGAAAAATTCGGCATCGGTGA | | | |  |
| primers for PLA qPCR (#60) | TTGCCTGACTGATCGCTAAA | GAGGGTTAGGGGAATCAAGG | | |  |
| DNA oligonucleotides for molecular cloning^3^ | | | | |  |
| TREX2_DMR | GAGGAATCCAGGGCCGTATG | CAGTAGGTGACAGGAGTGGG | | chrX:153444564-153445647 |  |
| TREX2_1 | ACCTGTGACCTTTGGCTCTG | GCCCAGAAGCCCAGGAAG | | chrX:153445597-153446046 |  |
| TREX2_PROM1 | CCACCACACCAGCTTACCT | AGCACCCCTTCCCCTTTG | | chrX:153445992-153446529 |  |
| TREX2_PROM2 | CCACCACACCAGCTTACCT | CTGTGAGCTAGCGAGGGG | | chrX:153445992-153446188 |  |
| TREX2_4 | CCCCTCGCTAGCTCACAG | ATTGACCCCAGGCCTGC | | chrX:153446171-153446336 |  |
| TREX2_5 | GCAGGCCTGGGGTCAAT | CTGCCCCATCCCTAGGTTTT | | chrX:153446320-153446554 |  |
| TREX2_6 | ATGAAGCGAGCTCTGGAGTG | GGTGACCCAGTATCTCCCTG | | chrX:153446457-153447285 |  |
| TREX2_7 | CCTACCAGTCACGCCTGTT | CTTTGGGGAGGGAGAGACAG | | chrX:153447203-153447730 |  |
| HAUS7_8 | CCAAGATGACAGGAGGGCC | ACACTCCCACCATCCTTTTG | | chrX:153447547-153447951 |  |
| CEBPB LAP^3^ | ATGCAACGCCTGGTGGCCTG | CTAGCAGTGGCCGGAGGAG | | chr20:50191034-50192071 |  |
| CEBPB LIP^3^ | ATGGCGGCGGGCTTCCCGTAC | CTAGCAGTGGCCGGAGGAG | | chr20:50191628-50192071 |  |
| CEBPA^3^ | ATGCGCGGGCGCGGGCGAG | TTATCACGCGCAGTTGCCCATGG | | chr19:33301338-33302518 |  |
| 6X CEBPA reporter | CTACCGTTGCACAATTACTATTGCGCAATTACCGTTTCGCAATTTGTATTGCGCAATTACCATTATGCAATTACTGTTCTGCAATTACGAGCT | | | |  |

^1^Primers were 5’-tagged with EpiTYPER tags (forward: AGGAAGAGAG, reverse: CAGTAATACGACTCACTATAGGGAGAAGGCT), ^2^Sequences were cloned into pGl4.10 or pGl4.23 using KpnI and XhoI (enhancer reporters) or BglII and HindIII (promoter reporters) restriction. Sequences were cloned into pCpGfree-promoter lucia using BamHI and SpeI restriction, ^3^Primers were 5’-tagged with Gateway homology sequences (forward: ggggacaagtttgtacaaaaaagcaggcttc, reverse: ggggaccactttgtacaagaaagctgggtt for insertion into Gateway donor vectors).
